# The NERD model: reflex circuit dysfunction as a systems-level driver of persistent post-concussion symptoms

**DOI:** 10.3389/fnsys.2025.1673195

**Published:** 2026-01-23

**Authors:** Matthew M. Antonucci, Kenneth Jay

**Affiliations:** 1 Department of Functional Neurology, Carrick Institute, Cape Canaveral, FL, United States; 2 Department of Neurology, Orlando College of Osteopathic Medicine, Winter Garden, FL, United States; 3 Department of Research, Cervello A/S, Roskilde, Denmark; 4 Department of Medicine, University of Central Florida, Orlando, FL, United States; 5 Department of Research, Ethos Health Group, Melbourne, FL, United States

**Keywords:** post-concussion symptoms, sensorimotor integration, gain modulation, cortical entrapment, network rigidity, neurorehabilitation, reflex circuits, functional neurology

## Abstract

Persistent post-concussive symptoms are often attributed to diffuse cortical dysfunction, yet this perspective may overlook key systems-level mechanisms. We propose a conceptual framework in which dysfunction arises from the dynamic interplay among five functional nodes: the sensory interface, reflex-brain stem hub, cerebellar module, basal ganglia–thalamic modulator, and cerebral cortex. Grounded in clinical observation and systems-level dynamic modeling, this framework treats the brain as a time-evolving control network that processes inputs, integrates them across hierarchical nodes, and generates adaptive or maladaptive outputs. Subcortical reflex circuits serve as critical nodes in the sensorimotor network, coordinating posture, orientation, and autonomic tone, and are modulated by cortical and thalamic systems. Injury to any of these nodes - or to the connections between them - can disrupt reflex control, distort afferent-efferent signaling, and compromise thalamocortical integration. The cerebellum calibrates predictive timing and coordination, while the basal ganglia-thalamic complex regulates gain and context-dependent gating. The cerebral cortex integrates intention, perception, and prediction to shape voluntary behavior and modulate reflex sensitivity. Reafferent feedback continuously updates the system, creating a dynamic loop of adaptation or maladaptation. Though this model has been applied clinically to guide early intervention, it remains a theoretical framework, untested by formal mathematical modeling or rigorous experimental validation. We offer it as a systems-level model that reframes post-concussive dysfunction as a network-level disorder, with reflex disintegration as a central, actionable mechanism.

## Introduction

Functional Neurology, as pioneered by Professor Carrick, is not rooted in theory alone - it is grounded in the systematic observation of clinical outcomes (Carrick and Carrick, 2020; Meyer et al., 2017). It began with a deceptively simple but powerful premise: observe the effects of neurological interventions in real patients, then model the mechanisms behind those effects to refine and replicate them. In this way, functional neurology rests on a foundation of clinically-driven hypotheses, continually tested and iterated in real-world settings (Meyer et al., 2017). While the clinical evidence base for functional neurology continues to develop through rigorous validation studies, its systems-oriented approach has demonstrated utility in the care of patients with complex neurological dysfunction, particularly those suffering from persistent symptoms after concussion (Francio et al., 2015; Swingen et al., 2017).

These cases resist reductionist explanations and demand a framework that can account for the dynamic interplay of reflexes, functional networks, behavior, and neuroplasticity. Too often, conventional paradigms attempt to localize dysfunction to static lesions or binary findings. Functional neurology, by contrast, embraces complexity; not as a problem to be simplified away, but as the terrain itself (Meyer et al., 2017). It offers a model, a language, and a therapeutic lens to engage with this complexity in a meaningful, reproducible, and actionable way. This systems-oriented clinical reasoning demands a new level of formalization; one that can articulate how disrupted networks evolve, compensate, and sometimes fail to recover without intervention.

Persistent symptoms following concussion pose substantial clinical challenges, significantly affecting patient quality of life and placing sustained burdens on healthcare systems worldwide (Iverson, 2019; Chandler et al., 2023; Jannace et al., 2023). Epidemiological studies indicate that 10–30% of individuals with concussion experience symptoms persisting beyond typical recovery windows, which average 2 weeks in adults and 4 weeks in children (Turner and LaBella, 2020). At one month post-injury, approximately 40–42% of patients report ongoing symptoms (Chandler et al., 2023), with measurable decrements in health-related quality of life and functional capacity. In military populations, up to 72% report at least one persistent post-concussive symptom, with prevalence ratios increasing with cumulative lifetime traumatic brain injuries (Jannace et al., 2023). Healthcare utilization patterns reveal substantial ongoing clinical needs, with many patients requiring extended follow-up care and demonstrating incomplete recovery trajectories (Tator et al., 2024; Ramsay et al., 2023). Although the pathophysiology of mild traumatic brain injury (mTBI) has been extensively characterized through neuroimaging, biomarker, and preclinical studies (Kenzie et al., 2017; Oft et al., 2024), clinical outcomes remain frustratingly inconsistent. Many individuals experience persistent symptoms that resist conventional rehabilitation (Leddy et al., 2016; Ellis et al., 2018; Ellis et al., 2015). This heterogeneity underscores a critical gap in mechanistic understanding, one that demands a model capable of explaining why a traditionally “transient” condition can lead to chronic, multisystem dysfunction, and why seemingly divergent therapeutic approaches may all yield benefit (Sullivan et al., 2023; Tiersen et al., 2023).

Acute concussion typically initiates a relatively consistent pathophysiological cascade involving multiple interconnected mechanisms. Primary mechanical forces produce axonal cytoskeletal disruption, microtubule remodeling, and impaired axonal transport (Peterson, 2022; Almenar-Queralt et al., 2022), leading to diffuse axonal injury that can persist chronically (Dikranian, 2019). Blood–brain barrier dysfunction occurs acutely and can remain detectable for months to years post-injury, with sensitive neuroimaging methods revealing sustained permeability and correlation with clinical outcomes (Liraz-Zaltsman et al., 2023; Heithoff et al., 2021). This barrier disruption triggers maladaptive astrocyte responses and facilitates neuroinflammatory cascades involving microglial activation, inflammasome signaling, and cytokine release (Wu et al., 2024; Green et al., 2024). Concurrently, metabolic derangements emerge, including glucose dysregulation, mitochondrial respiratory dysfunction, and oxidative modification of key proteins including mitochondrial complex I (Oft et al., 2024; Gowthami et al., 2023). These processes collectively produce non-specific neuronal depolarization, altered cerebral perfusion with hypoxia, cerebral edema, and eventual hypometabolism with disruption of network nodes (Kenzie et al., 2017). Among these nodes, the thalamus (Woodrow et al., 2023) and cerebellum (Mallott et al., 2019) reliably exhibit early connectivity disruption, often preceding the onset of post-concussive symptoms. Importantly, in the thalamus, these effects appear nucleus-specific (Woodrow et al., 2023), suggesting differential regional vulnerability not well accounted for by anatomical injury alone. While most individuals report symptom resolution within 30 days following concussion (Turner and LaBella, 2020; Chandler et al., 2023), a moderately powered longitudinal study using functional neuroimaging found that 83% of cases did not return to baseline brain function without intervention, despite clinical improvement in symptoms (Hiploylee et al., 2017). Recovery trajectories are highly heterogeneous, with distinct symptom phenotypes and temporal patterns emerging across individuals (Sullivan et al., 2023; Tiersen et al., 2023; Tator et al., 2024). This finding raises important questions about the nature of network reorganization, compensatory mechanisms, and the underlying drivers of persistent dysfunction (Gugger et al., 2022; Yang et al., 2021).

To address these limitations, we propose the Network Entrapment by Reflex Dysfunction (NERD) model, a dynamic systems framework that reframes persistent post-concussive symptoms as emergent phenomena. It conceptualizes the brain as a hierarchical, reflex-integrated network (Kaiser, 2008; Hütt et al., 2009) whose dysfunction arises from disinhibited feedback loops (Li et al., 2021; Sarma et al., 2021), degraded gain modulation (Wyrick and Mazzucato, 2020; Mejias et al., 2014; Stroud, 2018), and constrained adaptive capacity. Although the NERD model borrows conceptual language from network neuroscience - such as “hubs,” “nodes,” and “networks” - it is not a graph-theoretical model in the formal sense. We do not calculate metrics such as degree centrality or clustering coefficients, nor do we model probabilistic or topological path length. Instead, this framework is grounded in systems dynamics and control theory (Li et al., 2021; Sarma et al., 2021), emphasizing mechanistic feedback loops and state-dependent changes in functional capacity. The theoretical foundations draw from optimal control theory applied to sensorimotor systems (Li et al., 2021; Jagannathan and Xu, 2014), internal feedback architectures in biological control (Sarma et al., 2021), hierarchical brain organization principles (Kaiser, 2008; Hütt et al., 2009), and forward models with predictive coding (Mussa-Ivaldi and Fishbach, 2009; Bays, 2006; Idei et al., 2021). Neural network implementations of optimal feedback control provide computational frameworks for understanding how biological systems achieve adaptive motor and cognitive behavior under uncertainty (Jagannathan and Xu, 2014). The “network” described here refers to physiological circuits - vestibular, sensorimotor, cognitive, affective - whose interactions can be formalized using differential equations that evolve over time. Viewed this way, the brain becomes a dynamic hierarchy of interconnected nodes whose dysfunction unfolds through recursive feedback processes (Safari et al., 2020). To frame this complexity, the NERD model offers a systems-level explanation that links anatomical vulnerability, reflex disinhibition, and maladaptive reafferent signaling into a unified structure. In the following, we introduce its core architecture: five functional nodes that coordinate reflex modulation and network integration, a feedback loop that drives persistent symptom expression, and a visual map of how reflex disruption can entrap the brain in progressively rigid, low-adaptability states.

## The five functional nodes of the NERD model

The NERD model centers on five interdependent functional nodes, each representing a critical stage in the processing, modulation, and execution of sensorimotor and cognitive behavior. Dysfunction in any one node can degrade the system’s stability, but it is the recursive breakdown across nodes - via feedback loops and diaschitic effects - that leads to persistent symptoms.

### Sensory Interface (input + feedback node)

#### Structures

Peripheral receptors (muscle spindles, Golgi tendon organs, joint capsule mechanoreceptors, cutaneous receptors, baroreceptors, chemoreceptors, osmoreceptors, vestibular apparatus, cochlear hair cells, olfactory receptors, gustatory receptors, retinal photoreceptors); primary afferent pathways projecting via dorsal column-medial lemniscal, spinothalamic, spinoreticular, spinocerebellar, trigeminothalamic, and vestibular pathways; reafferent input generated by motor output via corollary discharge and efference copy mechanisms (Bays, 2006; Idei et al., 2021).

### Function

This node transduces and relays both incoming (exteroceptive and interoceptive) and internally generated (reafferent) sensory signals. It anchors the system to the body and environment, providing raw inputs for reflex arcs, cerebellar calibration, and cortical prediction. Efference copy mechanisms enable predictive attenuation of self-generated sensations, a process that develops through sensorimotor experience and free-energy minimization (Bays, 2006; Idei et al., 2021). Disruption of sensory feedback pathways or reafferent signal processing can distort the brain’s internal representation of body state, leading to maladaptive motor adjustments and impaired predictive control.

### Reflex-brainstem hub (foundational response node)

For clarification, Jackson (Jackson, 1884) have used ‘primitive reflexes’ to describe foundational reflex circuits, but because the term now refers to specific developmental reflexes (e.g., ATNR), we use each term in its proper context, defining foundational reflexes as semi-autonomous sensory-motor circuits spanning simple responses like the VOR to more complex patterns like the ATNR.

#### Structures

Vestibular nuclei, Reticular formations, Superior colliculus, Inferior colliculus, Periaqueductal gray (PAG), Pretectal area, Tegmentum, Red nucleus, Parabrachial nucleus, Nucleus tractus solitarius (NTS), Cranial nerve nuclei, Medullary cardiorespiratory centers, Pontine reflex centers, Spinal interneurons, Central pattern generators (CPGs), Dorsal horn reflex pathways.

#### Function

Executes low-latency reflexes essential for posture, gaze, orientation, and autonomic control (Robinson, 2022; Tarnutzer and Palla, 2013). This hub provides rapid, patterned responses and sets baseline gain for motor outputs, shaped by inputs from cerebellum, cortex, and basal ganglia. The vestibulo-ocular reflex (VOR) exemplifies this node’s function, implementing rapid gaze stabilization through canal biophysics, push-pull organization, velocity-storage mechanisms, and neural integration (Robinson, 2022). VOR gain can be adaptively modified through visual-vestibular interactions and cross-modal plasticity involving semicircular canal and otolith signals (Paige and Fredrickson, 1990; Koizuka et al., 2004). The VOR interacts nonlinearly with smooth-pursuit systems, with pursuit compensating residual VOR errors during visual fixation (Bock, 1984). Following unilateral vestibular deafferentation, central compensation mechanisms restore dynamic function and reduce static symptoms over time through bilateral balance recalibration (Tarnutzer and Palla, 2013). Disruption of these reflex circuits can lead to disinhibition of primitive reflexes that normally remain suppressed in adults. Evidence from Parkinson’s disease demonstrates that loss of higher cortical and subcortical inhibition permits re-expression of developmentally primitive motor programs via subcortical disinhibition (Vreeling et al., 1993). Similarly, in cerebral palsy and autism spectrum disorders, delayed disappearance or persistence of primitive reflexes correlates with compromised cortical control and altered long-range connectivity patterns (Çocuk Nörolojisi, 2016; Melillo et al., 2023). High-risk neonates with perinatal injury show variable primitive reflex integrity, suggesting that network abnormalities determine whether primitive reflexes are expressed or suppressed (Sohn et al., 2011).

### Cerebellar module (predictive calibration node)

#### Structures

Cerebellar cortex (vermis, intermediate zone, lateral hemispheres, flocculonodular lobe); deep cerebellar nuclei (fastigial, interposed, dentate, vestibular nuclei); input relays from the inferior olivary nucleus and pontine nuclei; output projections via the superior and inferior cerebellar peduncles; cerebellar afferent pathways (spinocerebellar, cuneocerebellar, vestibulo cerebellar, and pontocerebellar tracts).

#### Function

Calibrates motor output by implementing forward models and temporal predictions (Arbib and Spoelstra, 1999; Mussa-Ivaldi and Fishbach, 2009). Modulates brainstem reflexes and enhances thalamic relay precision, enabling adaptive motor control and temporal coherence in behavior. Purkinje cell populations in the vestibulo-cerebellum encode sensory prediction errors, with distinct populations representing tilt velocity and linear acceleration consistent with forward model computations requiring temporal integration (Laurens and Angelaki, 2019). The cerebellar module learns forward and inverse models using eligibility traces to solve temporal credit-assignment problems and compensate for sensorimotor delays (Arbib and Spoelstra, 1999). Spiking cerebellar circuit implementations with predictive learning rules reproduce multi-timescale adaptation phenomena including learning curves, savings, and aftereffects observed in human motor adaptation (Wu et al., 2021). The cerebellum implements an internal clock for timing in the 10–100 ms range, encoding elapsed time and providing gain and timing control for motor and cognitive tasks (Yamazaki, 2012). The cortico-deep cerebellar nuclei loop implements prediction (cortex) and filtering/integration (nuclei), with distributed plasticity allowing compensation and reserve capacity after damage (Mitoma et al., 2023). This architecture supports the cerebellar node’s role in error-based learning, predictive timing, and resilient predictive computations essential for NERD’s adaptive control mechanisms.

### Basal ganglia-thalamic modulator (state-based gating node)

#### Structures

Striatum (caudate nucleus, putamen, nucleus accumbens), globus pallidus (external and internal segments), subthalamic nucleus (STN), substantia nigra (pars compacta and pars reticulata), thalamic relay nuclei (ventral anterior [VA], ventral lateral [VL], centromedian and parafascicular intralaminar nuclei), and associated cortical input/output loops.

#### Function

Filters and gates ascending and descending information based on context and relevance (Wang and Halassa, 2021; Mulcahy et al., 2020). Sets the gain for motor and cognitive loops by modulating thalamocortical relay via tonic inhibition, thereby regulating intentionality, timing, and effort allocation. Thalamocortical interactions serve meta-learning and credit-assignment functions, with the thalamus providing cortical control functions that basal ganglia select across timescales (Wang and Halassa, 2021). Anatomically, GABAergic basal ganglia outputs (GPi/SNr) converge onto single motor thalamic neurons together with excitatory inputs from M1 layer 5 and cerebellum, providing a substrate for BG output to directly control thalamic relay gain for cortical and cerebellar information streams (Koster and Sherman, 2024). Computational models demonstrate that the BG-thalamo-cortical loop implements corticostriatal potentiation for learning, BG re-engagement during reversal learning, and can produce pathological oscillations that disrupt action selection when dysregulated (Mulcahy et al., 2020). Optogenetic studies confirm that transient inhibition or excitation of SNr outputs to motor thalamus bidirectionally biases directional choices and that tonic nigral output suppresses unwanted movements, providing causal evidence for BG gating of action initiation and decision bias (Morrissette et al., 2018). The rostral intralaminar nuclear complex (rILN) projects to dorsal striatum, signaling at action initiation and reward acquisition, with optogenetic manipulation of this pathway bidirectionally altering task performance and linking thalamic input to striatal reinforcement-driven action selection (Cover et al., 2022). These mechanisms position the basal ganglia-thalamic node as a critical controller of context-dependent gating, gain modulation, and action selection within the NERD framework.

### Cerebral cortex (executive control node)

#### Structures

Includes primary sensory areas (Primary Somatosensory Cortex [S1], Primary Visual Cortex [V1], Primary Auditory Cortex [A1], Primary Olfactory Cortex, Primary Gustatory Cortex, Vestibular Cortex) and their secondary and association areas (Secondary Somatosensory Cortex [S2], Visual Association Cortices [V2, V3, V4, V5/MT], Auditory Association Cortex, Olfactory and Gustatory Association Cortices, Posterior Parietal Cortex, Superior and Inferior Temporal Gyri, Parietal Operculum, Insular Cortex).

Motor-related regions include the Primary Motor Cortex (M1), Premotor Cortex (PMA), Supplementary Motor Area (SMA), Pre-Supplementary Motor Area (pre-SMA), Frontal Eye Fields (FEF), and Broca’s Area.

Prefrontal regions include the Dorsolateral Prefrontal Cortex (DLPFC), Ventrolateral PFC (VLPFC), Orbitofrontal Cortex (OFC), Ventromedial PFC (vmPFC), Dorsomedial PFC (dmPFC), Medial PFC (mPFC), Anterior PFC (Frontal Pole), Inferior, Middle, and Superior Frontal Gyri.

Limbic and paralimbic regions include the Cingulate Gyrus (Anterior and Posterior Cingulate Cortex), Parahippocampal Gyrus, Entorhinal Cortex, Perirhinal Cortex, Subiculum, Presubiculum, Hippocampus, Retrosplenial Cortex, Temporal Pole, Amygdala, and overlapping hubs such as the OFC, mPFC, and Insular Cortex.

Additional integration hubs include the Temporoparietal Junction (TPJ) and Precuneus.

#### Function

The cerebral cortex integrates multisensory input to generate behavioral plans, predictive models, and voluntary control over action and cognition. It serves as a top-down regulatory system that guides perception, attention, intention, and decision-making. Through widespread projections to subcortical structures (e.g., cerebellum, basal ganglia) and brainstem nuclei, it modulates reflex circuits and incorporates feedback to support adaptive, goal-directed behavior. Following traumatic brain injury, large-scale cortical network disruption is well-documented. Structural connectome deviations persist after mTBI, correlate with blood biomarkers of axonal and glial injury (GFAP, NfL), and concentrate in structural network hubs, with the magnitude of network abnormalities determining post-injury recovery trajectories (Gugger et al., 2022). Functional connectivity studies reveal heterogeneous but consistent alterations involving the default mode network, salience and attention networks, and whole-brain connectivity with time-dependent patterns including early decreases and variable later changes (Dogra et al., 2024). Animal models demonstrate rapid compensatory reorganization after TBI, including contralesional increases in node strength, higher modularity with influential nodes confined to contralesional modules, and persistent ipsilateral deficits (Yang et al., 2021). At the cellular level, somatostatin interneurons become hyperconnected local hubs with reduced long-range inputs after TBI, implying loss of distal inhibitory control despite preserved local connectivity capacity (Lau and Lillis, 2023). Homeostatic rewiring near injury sites produces hyperconnected local subnetworks with preserved E/I balance but lower burst thresholds and propensity to initiate network bursts, demonstrating how maladaptive local hyperconnectivity and impaired long-range regulation can produce aberrant subcortical circuit activation (Ghiasvand et al., 2022). These network-level changes provide the substrate for cortical entrapment and loss of top-down control over reflex circuits central to the NERD model.

## Core feedback loop: from reflex injury to network rigidity

The NERD model describes a cascading feedback loop in which initial injury to reflex-modulating structures - such as the brainstem, midbrain, cerebellum, or thalamus - leads to maladaptive motor output. This output generates distorted reafferent sensory signals, which further degrade gain modulation and overload thalamocortical/corticothalamic systems. As this loop recycles, the brain’s ability to regulate input, filter salience, and switch efficiently between networks becomes increasingly compromised. The result is progressive network rigidity, reflected in cognitive fatigue, sensorimotor disintegration, autonomic dysregulation, and behavioral inflexibility. This loop is not linear but recursive - each output becomes new input, anchoring the system in a maladaptive attractor state unless interrupted by intervention.

## Visualizing entrapment: the NERD spiral diagram

**Figure 1:**
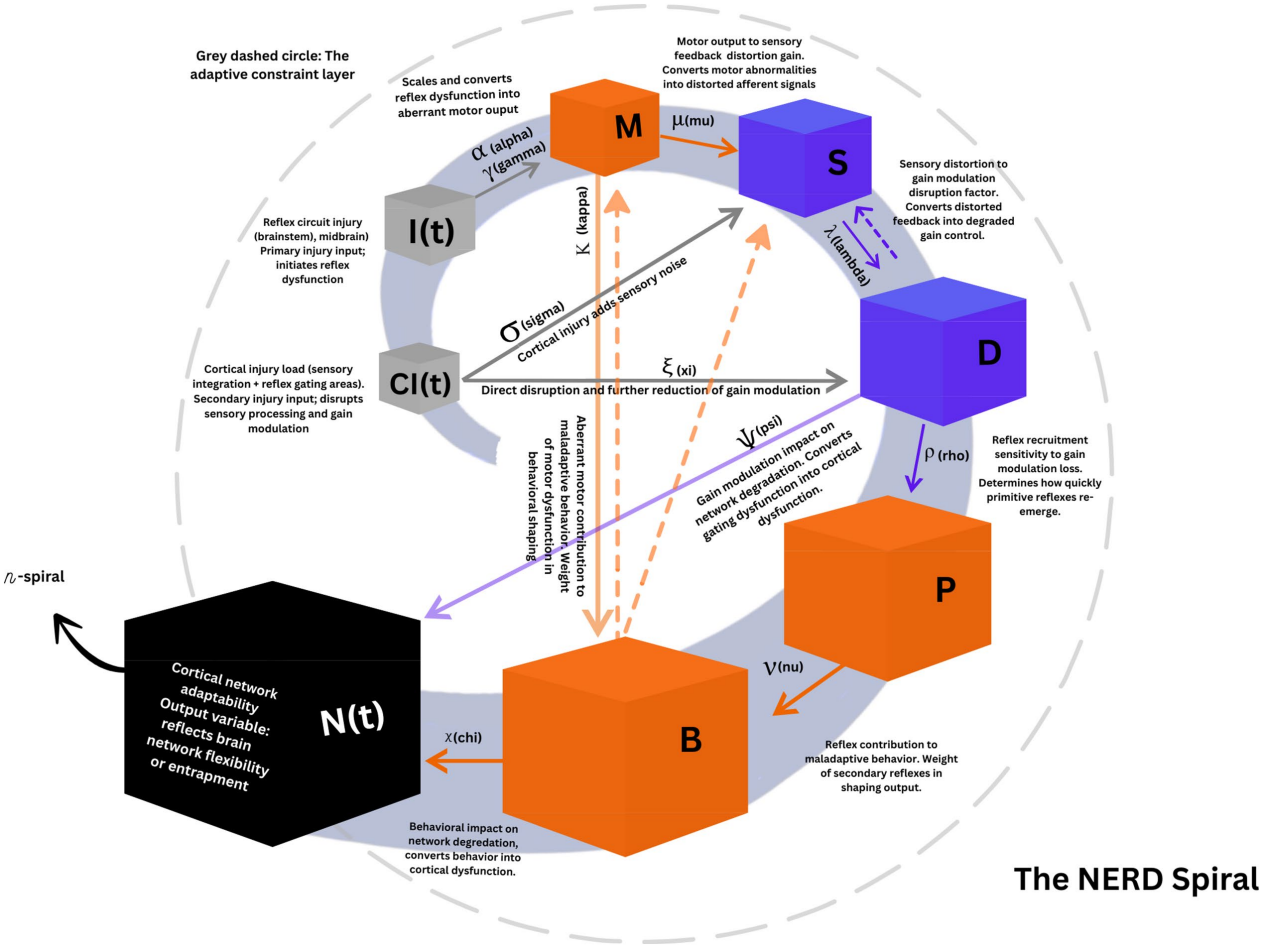
The NERD spiral – visual mapping of model dynamics. The outward-winding coil depicts the temporal cascade from acute injury to cortical entrapment. Each turn corresponds to a new stage in the chain; cube size increases with radius to convey greater downstream impact. (1) Seed injuries (grey cubes). Reflex-circuit damage I(t) and cortical injury C_I_(t) sit at the innermost turn. Injury to the reflex arc is weighted by the vulnerability product *γα* and immediately up-scales the motor-output node M (grey arrow). (2) Dual divergence from M. Exaggerated motor drive propagates toward maladaptive behavior (B) via the weight *κ* (orange). The same motor activity injects noise into the sensory-distortion node (S) via *μ* (orange). (3) Cortical injury inputs. C_I_(t) adds sensory noise at S (*σ*) and directly reduces the gain-control capacity (D) (grey → blue) through the coefficient *ξ*. (4) Mid-level feedback loop. Sensory distortion and gain loss reinforce each other: S → *λ* → D (blue) while diminished gain recruits additional reflex pathways: D → ρ → P (blue), and those reflexes shape behavior: P → *ν* → B. (5) Convergence on cortical adaptability. The two colored edges (*ψ* and χ) meet their vertices at N(t), orange via the behavioral-impact gain *χ* [B → N(t)] and blue via the gating-impact gain *ψ* [D → N(t)]. (6) Spiral of decompensation. Once the combined load reaches N(t), the black *η*-spiral takes over: η integrates both stresses over time, widening each subsequent coil and signalling that adaptation has given way to progressively rigid network dynamics. Dashed arrows (orange and blue) represent recursive feedback loops that can either sustain existing dysfunction or, in some cases, initiate maladaptive dynamics. These loops reflect the system’s capacity for self-reinforcing behavioral, sensorimotor, or gating-based adaptations that may evolve into primary drivers of network entrapment. The black dashed circular boundary represents the adaptive constraint layer: a combination of environmental, structural, and psychological buffering mechanisms that initially contain reflex-driven dysfunction. In accordance with allostatic principles, this constraint layer is not static; it can adjust to a new setpoint over time, re-stabilizing the system even in maladaptive or inefficient states. Thus, the model does not predict inevitable collapse, but conditional containment or entrapment depending on the interplay between loop amplification and constraint adaptation. NERD-equation congruence. Every coefficient or variable that appears in the differential NERD equation is rendered in the visual presentation of the model, as a colored arrow, a labelled cube, or the black spiral. The sole implicit quantity is the reference reserve G_0_. It is not drawn as its own box because it functions as a baseline gain reserve, visually suggested by the distance between the gain-loss hub D and the adaptability node N(t). Finally, the outward expansion of the spiral embodies the negative sign and the global scaling factor η in dN/dt: once the combined orange + blue stresses reach N(t) entrapment accelerates outward. While the cascade often begins with reflex circuit injury [I(t)], cortical injury [CI(t)] may also initiate the process by disrupting perceptual integration or behavioral regulation, such as through impaired visual input, thereby indirectly loading reflex circuits and degrading gain modulation over time.

The dynamic feedback loop described by the NERD model is captured visually in the NERD Spiral - a conceptual diagram illustrating how initial injury evolves into widespread network dysfunction. At the center are seed injuries to reflex circuits and cortical integration areas. These injuries initiate exaggerated motor output, which produces distorted sensory feedback. As gain modulation deteriorates, primitive reflexes are recruited, maladaptive behaviors emerge, and both reflexive and cortical regulation decline. The spiral widens with each turn, symbolizing the increasing load on network adaptability. Once the system’s plastic reserve is exceeded, it becomes trapped in a progressively rigid state. This spiral representation underscores the model’s central claim: that reflex dysfunction can anchor a self-perpetuating cycle of degradation unless specifically interrupted (Figure 1).

## Current state of the field

Despite advances in pathophysiological understanding, clinical models of concussion continue to emphasize transient cortical disruption and early metabolic cascades (Patricios et al., 2023; Quatman-Yates et al., 2016). While this framework explains many acute symptoms, it offers limited insight into the chronic, multisystem dysfunction observed in a subset of patients. This conceptual gap has contributed to persistent inconsistencies in diagnosis, rehabilitation strategies, and long-term outcomes.

Emerging perspectives in network neuroscience suggest that concussion should be framed less as a focal injury and more as a disturbance of distributed brain systems, particularly those involved in sensory integration, reflex modulation, and large-scale network coordination (Sharp et al., 2014; Mayer et al., 2011, 2012). While reflex circuit dysfunction is increasingly acknowledged in research circles, clinical practice remains largely anchored in symptom-based frameworks that fail to operationalize these insights into diagnostic or therapeutic strategy. When disrupted by anatomical injury, these simple sensorimotor integrations produce abnormal outputs that distort sensory feedback, impair gating, and destabilize network coordination, yet they remain largely absent from diagnostic and rehabilitative strategies.

Evidence from neuroimaging has consistently shown persistent alterations in brain network dynamics among individuals with chronic mTBI. Graph theory–based analyses indicate that these individuals often exhibit reduced modularity, increased local clustering, and impaired transitions between large-scale networks, functional signatures associated with cognitive rigidity, postural asymmetry, and dual-task performance deficits (Hillary et al., 2014; Wang et al., 2018; D’Souza et al., 2020). While such patterns have historically been attributed to diffuse axonal injury or metabolic derangement, a growing body of research points toward the breakdown of reflex gating and cross-network modulation as a plausible mechanism (Mayer et al., 2011; Sherman, 2016).

Importantly, concussion-related impairments do not occur in isolation from other neurophysiological systems. Comorbid conditions such as post-traumatic stress disorder (PTSD) often present with overlapping disruptions in limbic, salience, and sensorimotor networks, suggesting a shared vulnerability in subcortical nodes that regulate arousal, threat processing, and motor readiness (Palacios et al., 2013; Cisler et al., 2014). These converging network disruptions support a transdiagnostic model in which maladaptive reflex anchoring and impaired sensorimotor gating reflect common pathophysiological substrates.

Despite increasing recognition of these systems-level disturbances, the clinical implementation of reflex-based assessment and intervention remains limited. Reflexive behaviors, including those retained from early development, are rarely evaluated in adult neurological exams - despite evidence that developmental reflexes, once thought to extinguish in early life, can re-emerge in post-concussive states as a sign of subcortical disinhibition (Zafeiriou, 2004; Wortzel et al., 2009). Their dysregulation may contribute significantly to sensorimotor noise, postural inefficiency, and cognitive overload in chronic mTBI populations.

The field currently lacks a unifying mechanistic framework that connects these reflex-level disruptions to large-scale network dysfunction. Existing models often treat symptoms such as dizziness, cognitive fatigue, and emotional lability as parallel sequelae of a single injury event rather than interacting outputs of disrupted neural systems. In doing so, they fail to account for the network rigidity, sensorimotor disintegration, and reflex dominance that can emerge when inhibitory control from higher-order centers is lost.

In light of these gaps, there is increasing momentum toward models that incorporate reflex physiology, diaschitic effects, and network neuroscience to explain the chronicity and heterogeneity of post-concussive syndromes. A growing number of clinicians and researchers are recognizing that subcortical circuits, once considered primitive or peripheral, may be central to both the expression of symptoms and the failure of recovery. Yet formal integration of these insights into clinical frameworks, rehabilitation algorithms, and mechanistic models remains in its infancy.

## From hub disruption to reflex dominance: a systems neuroscience perspective

As previously outlined in the NERD model’s architecture, the cerebellum and thalamus function as high-order integrative hubs that coordinate multisensory processing, reflex modulation, and network switching. When disrupted, either by direct injury or diaschitic effects, their loss of inhibitory control over subcortical reflex circuits can trigger a cascade of maladaptive reorganization, leading to a shift toward more foundational neural control patterns (Sherman, 2016; Woodrow et al., 2023)(Chen et al., 2022) (L. Zhang et al., 2004; J. Zhang et al., 2016).

From a systems neuroscience perspective, diaschisis, the dysfunction of remote but connected regions following focal injury, offers a plausible mechanism. When higher-order “node” structures like the thalamus or cerebellum are impaired, their regulatory control over subcortical reflex circuits diminishes. In line with Jacksonian dissolution (Jackson, 1884), this loss of cortical inhibition leads to a regression toward more reflexive behaviors, a pattern observed in many chronic mTBI cases.

- Key Point: Jacksonian dissolution, proposed by Hughlings Jackson in the late 19th century, describes the regression to more primitive neural functions when higher cortical control is lost (Jackson, 1884). In this view, damage to integrative centers leads to disinhibition of lower-level reflexes, resulting in exaggerated or re-emergent motor patterns. This model remains relevant today as a framework for understanding the reflex dominance and sensorimotor rigidity observed after concussion.

Despite increasing recognition of concussion as a network-level disorder, reflex circuits - especially those involving cervical afferents and primitive/postural responses - remain under-recognized in both research and clinical frameworks. Yet these circuits have the capacity to destabilize higher-order brain networks through maladaptive feedback, acting as diaschitic drivers of global dysfunction when left unmodulated (Mayer et al., 2011; Sharp et al., 2014).

## Evidence of structural vulnerability after concussion

The brain’s anatomical architecture renders certain midline structures, particularly the brainstem, midbrain, thalamus, and cerebellum, especially vulnerable to concussive shear forces, even in cases classified as “mild” traumatic brain injury. These regions lie at the interface between mobile and tethered tissue, making them susceptible to rotational strain and microstructural injury (Gentry, 1994; Margulies and Thibault, 1992), and thalamo-cortical communication, even in the absence of overt parenchymal injury (Hulkower et al., 2013; Messé et al., 2011). Importantly, they house the reflex-modulating and gain-regulating hubs identified in the NERD model, including the vestibular nuclei, deep cerebellar nuclei, and thalamic relay centers.

Among these, the thalamus is one of the most consistently affected regions following concussion. Injury to relay nuclei such as the pulvinar, mediodorsal, and intralaminar groups disrupts thalamocortical feedback, sensory prioritization, and cognitive integration, core processes tied to network flexibility and attentional control (Llinás et al., 1999; Sherman, 2016; Mayer et al., 2011). Similarly, midbrain structures like the tectum, periaqueductal gray, and red nucleus, which are essential for reflex modulation and sensorimotor coordination, are biomechanically vulnerable and frequently exhibit dysfunction after concussion (Taylor and Gandevia, 2004; Blythe, 2005).

Structural imaging studies corroborate these patterns, showing consistent changes in thalamic, cerebellar, and brainstem regions across modalities and populations (Hillary et al., 2014; L. Zhang et al., 2004; J. Zhang et al., 2016; D’Souza et al., 2020). These anatomical disruptions impair the brain’s ability to calibrate reflex gain, suppress maladaptive motor patterns, and maintain adaptive cross-network signaling. Table 1 below summarizes the biomechanical vulnerability, functional roles, and clinical consequences of key subcortical structures involved in reflex modulation and network regulation. Together, these nodes form the anatomical substrate through which the NERD model operates.

**Table 1:** Structural vulnerability and functional roles of subcortical reflex-modulating nodes affected by concussion.

Hub/structure	Biomechanical vulnerability	Primary functional role	Consequences of injury
Brainstem	Very High (rotational shearing to deep midline structures; tensile strain on cervical spine; DAI)	Regulate vital functions, consciousness and wakefulness, sensory-motor relay, basic reflexes, posture, balance, cortico-cerebellar connection	Dysautonomia, altered consciousness, cranial nerve dysfunction, sensory and auditory impairment, dizziness, vertigo, balance, fatigue, cognitive/affective disorders, pain processing, tone generation
Midbrain	High (rotational shear at brainstem- diencephalon junction)	Reflex inhibition, motor readiness, arousal modulation, ocular alignment/movement, sensory-motor integration.	Dysfunction of foundational reflexes (eg: pupillary light reflex, accommodation reflex, and primitive reflexes) causing, postural instability, light sensitivity, blurred vision, slowness, sleep disorders,
Cerebellar peduncles	High (white matter tracts prone to shear)	Bidirectional communication (but largely efferent projections) between cerebellum, thalamus, cortex	Disrupted cerebello-thalamo-cortical signaling, impaired sensorimotor timing
Cerebellum (deep nuclei)	Moderate (dense architecture, higher cell content, more connections, foramen magnum, but less mobile)	Modulation of vestibular/postural reflexes, coordination, feedback/feedforward/efferent copy	Gaze instability, dysmetria, gait changes, oculomotor dysfunction, affect/cognition changes, impaired predictive control
Thalamus/hypothalamus/pituitary/ amygdala/hippocampus	High (central hub at white-gray matter junctions, in sheer planes with proximity to bony structures)	Sensory gating, cross-network switching, cognitive integration, circadian rhythms, memory, limbic functions, autonomic, neuro-endcorine	Impaired attention, network rigidity, sensory-motor disintegration, insomnia, somnolence, hormonal dysfunction, amnesia, impaired emotional responses

This table summarizes key subcortical structures vulnerable to biomechanical injury and central to reflex modulation and network control. These nodes - including the midbrain, thalamus, brainstem nuclei, and deep cerebellar circuits - regulate reflex gain, sensory gating, and multisystem coordination. When disrupted, they contribute to maladaptive feedback loops and network rigidity, as described in the NERD model.

## Toward a systems-level understanding of reflex dysfunction in concussion

Sensory integration is a hierarchical process composed of five stages: registration, orientation and attention, interpretation, organization of a response, and execution. As first proposed by Ayres and Robbins (2005), this framework highlights how the brain transforms sensory input into coordinated motor output through increasingly complex integrative layers (Ayres and Robbins, 2005).

- Key Concept: Sensory integration, as originally described by Ayres and Robbins (2005), unfolds through a five-stage process: sensory registration, orientation and attention, interpretation, organization of a response, and motor execution. Each stage builds upon the last, requiring precise input and efficient central processing to generate adaptive behavior. Disruption at any of these stages, particularly at the foundational levels of sensory registration and orientation, can compromise all downstream processes. In the context of concussion, reflex circuit dysfunction undermines this hierarchy at its base, destabilizing the sensorimotor scaffolding upon which higher-order cognitive and affective functions depend (Ayres and Robbins, 2005).

The National Institutes of Health has emphasized that a substantial portion of the brain’s metabolic and structural resources are devoted to processing sensory input in the service of adaptive behavior (National Institutes of Health and Biological Sciences Curriculum, 2007). In this model, both sensory precision and integrative capacity determine the quality of motor output; put simply, the brain’s ability to act depends on its ability to perceive and integrate.

Concussion frequently disrupts reflex systems at the brainstem and cerebellar levels. Among the most commonly impaired are the vestibulo-ocular reflex (VOR) and vestibulospinal reflex (VSR), which regulate spatial orientation and postural control (Gard et al., 2022; Crampton et al., 2021). The cervico-ocular reflex (COR) compounds visual–spatial disorientation (Kristjansson and Treleaven, 2009), while the acoustic startle reflex (ASR), pupillary light reflex (PLR), baroreceptor reflexes, blink reflexes, and gag reflexes reflect broader subcortical dysregulation (Liska et al., 2018; McKee et al., 2024; Hsu et al., 2021; Hilz et al., 2016; La Fountaine et al., 2022). Notably, these reflexes share common integration centers in the brainstem and cerebellum, regions especially vulnerable to concussive shear forces. Their dysfunction signals a breakdown in reflex gain calibration and reafferent filtering, undermining multisensory precision (Zhang et al., 2004). From a developmental perspective, reflex circuits are among the earliest systems to emerge in the embryonic nervous system. Vestibular reflexes, for example, are present by 12 weeks post-conception and respond to gravitational input and early movement (Walther, 2005; Ellis et al., 2018). These primitive systems form the scaffolding for higher-order cortical development - not merely preceding it, but shaping it. Injury to subcortical nodes housing these reflexes threatens the foundation of multisensory integration and adaptive network flexibility (Previc, 1990; Blythe, 2005; Hadders-Algra, 2005).

This systems-level perspective reframes concussion pathology: rather than a condition of diffuse or focal cortical dysfunction alone, it is a disorder of maladaptive feedback loops anchored in dysregulated subcortical reflex networks. These circuits, when impaired or disinhibited, may act as diaschitic drivers, destabilizing distributed networks and constraining the brain’s capacity for adaptive response.

## Functional reflex disruption

Multiple reflex systems, primarily subcortical and mediated by brainstem or cerebellar pathways, demonstrate measurable dysfunction following concussion. These reflexes, while often described as simple sensorimotor loops, play essential roles in gaze stabilization, postural control, autonomic regulation, and sensory integration. Their impairment offers a clinically observable proxy for instability in subcortical integration centers and disrupted top-down modulation.

Among the most commonly affected are the vestibulo-ocular reflex (VOR) and vestibulospinal reflex (VSR), which help maintain spatial orientation and postural stability through coordinated brainstem and cerebellar activity (Gard et al., 2022; Crampton et al., 2021). The cervico-ocular reflex (COR), which links cervical afferents to gaze control, is also frequently disrupted, contributing to disorientation and visual instability (Kristjansson and Treleaven, 2009). Autonomic and cranial reflexes such as the pupillary light reflex (PLR), acoustic startle reflex (ASR), blink reflex, and gag reflex further illustrate the extent of subcortical disruption. These reflexes may present with slowed, exaggerated, or asymmetric responses - markers of altered excitability and brainstem dysregulation (McKee et al., 2024; Hsu et al., 2021; Hilz et al., 2016; La Fountaine et al., 2022; Liska et al., 2018; Gard et al., 2022; Crampton et al., 2021).

These reflexes share common neuroanatomical integration in the brainstem and cerebellum - regions known to be especially vulnerable to rotational injury and shear forces (Table 2). Their dysfunction is not epiphenomenal but reflects core breakdowns in reflex gain modulation, suprasegmental gating, and multisensory coordination. The high prevalence of vestibular and cranial reflex impairment in post-concussive populations underscores their diagnostic and functional relevance (Gard et al., 2022).

**Table 2:** Foundational Reflex Domains and Clinical Relevance.

Category	Reflexes/circuits	Primary function	Neuroanatomical integration	Clinical implications
Vestibulo-Ocular	VOR, OTR	Gaze stabilization during head movement	Vestibular nuclei, cerebellum (flocculus), oculomotor nuclei	Oscillopsia, blurred vision, visual vertigo
Vestibulo-Cervical-Spinal	VCR, CCR, COR, VSR	Head–gaze–posture coordination; tone modulation	Vestibular nuclei, cerebellum (nodulus), cervical afferents, cerebellum	Dizziness, neck stiffness, spatial disorientation
Postural/Righting	Labyrinthine Righting, Optical Righting, Ocular Tilt	Orientation to gravity and visual horizon	Vestibular nuclei, midbrain, brainstem cerebellum	Postural misalignment, poor spatial referencing
Autonomic-Linked	VSR, PLR, Baroreceptor Reflex, chemoreceptor	Autonomic adaptation to light, movement, respiration, and posture	Brainstem, PAG, amygdala, hypothalamus	Orthostatic intolerance, HRV instability, apnea
Cranial Defensive	Blink, Gag, Acoustic Startle (ASR)	Protective reflexes; arousal modulation	Pons, medulla, midbrain (PAG)	Hypersensitivity, startle, nausea, gag dysfunction, anxiety
Primitive	ATNR, STNR, TLR, Moro	Early motor patterning	Cerebellum, cortical circuits	Re-emergence linked to postural/cognitive rigidity
Central Modulators	PAG, Red Nucleus, Tectum, Thalamus	Reflex gain modulation, multisensory weighting	Midbrain, thalamus, hypothalamus	Reflex disinhibition, affective/autonomic lability
Network Expression	DMN, SN, FPN, SMN, DAN,	Large-scale network flexibility and integration	Cortico-subcortical networks	Cognitive fatigue, dual-task intolerance, rigidity, sensory-motor processing, attention

This table categorizes reflex systems involved in sensorimotor coordination, autonomic regulation, and multisensory integration. Each reflex group - vestibulo-ocular, postural, autonomic-linked, cranial, primitive, and modulatory - shares integration points in subcortical hubs vulnerable to concussion. Dysfunction in these circuits contributes to impaired sensory gating, maladaptive motor responses, and reduced network flexibility. The final row links these reflex domains to large-scale cortical networks, emphasizing how reflex disintegration can propagate to higher-order cognitive, affective, and attentional systems, as described in the NERD model. The table provides a conceptual foundation for understanding how disturbances across these domains may contribute to persistent post-concussive symptoms affecting posture, vision, autonomic control, cognition, and emotion.

Viewed through a systems-neuroscience lens, reflex impairment signifies more than a local deficit: it represents a failure of dynamic calibration across distributed networks. When brainstem and cerebellar reflex loops are damaged or disinhibited, they can propagate distorted afferent signals upward, destabilizing sensorimotor integration and anchoring maladaptive network states. These changes manifest not only in motor symptoms but also in cognitive, affective, and autonomic domains, revealing reflex dysfunction as a diaschitic driver of multisystem impairment following concussion.

Although we emphasize vestibular and autonomic reflex circuits as primary illustrations of the NERD model in the present paper, the framework extends in full to somatosensory, visual, and cognitive systems. These domains, while not elaborated in detail here, are governed by the same hierarchical control architecture, recursive feedback loops, and gain modulation principles that underlie the model’s core dynamics. Disruptions in proprioceptive integration, visual weighting, or cognitive adaptability are not isolated phenomena but reflect network-level consequences of reflex circuit instability and degraded thalamocortical coordination. Their inclusion within the model is essential and remains grounded in the same systems-level mechanisms articulated throughout.

## Reflex disruption as a bidirectional diaschitic mechanism

Persistent post-concussive symptoms remain difficult to resolve in part because existing models fail to capture the systems-level and network-based dysfunction that underlies chronic mTBI (Patricios et al., 2023; Quatman-Yates et al., 2016). The reflex impairment framework addresses this gap by positioning reflex circuit disruption, whether through direct structural injury or suprasegmental disinhibition, as a bidirectional diaschitic mechanism that drives and sustains network dysregulation.

- Key concept: First described by Monakow in 1914, diaschisis refers to reduced function in regions remote from primary injury due to disrupted afferent input. While originally conceived as a purely inhibitory phenomenon, modern interpretations recognize that diaschisis can also manifest as hyperactivity, driven by disinhibition, maladaptive plasticity, or compensatory overdrive (Carrera and Tononi, 2014).

In the healthy brain, subcortical reflexes are regulated by descending projections from the cortex, midbrain, cerebellum, and thalamus. Midbrain structures like the periaqueductal gray, tectum, and red nucleus provide top-down inhibition to reflex generators in the brainstem and spinal cord, allowing reflex gain to adapt to context. Thalamic relay nuclei also shape reflex sensitivity by integrating multisensory input and predictive signals from the cortex and cerebellum (Llinás et al., 1999; Cavanna and Trimble, 2006).

The cerebellum plays a particularly central role in reflex gain modulation, especially for the vestibulo-ocular reflex (VOR) and vestibulospinal reflex (VSR), through cerebellar floccular and nodular function, and fastigial outputs to the vestibular nuclei and brainstem. These projections enable adaptive calibration of eye movements, balance, and postural tone in response to environmental and internal demands. Similarly, the reticular formation sets the baseline excitability of alpha and gamma motor neurons, effectively tuning global muscle tone and influencing the gain of spinal reflex arcs. Damage to these structures disrupts this calibration, promoting either hypo- or hypertonia, dysmetria, or ataxia (of motor, cognition, or affect) depending on the nature of the dysregulation (Rudolph et al., 2023).

- Key Point: The cerebellum modulates state-dependent gain across motor, cognitive, affective, and autonomic systems through continuous integration of bottom-up and top-down inputs. Spinocerebellar and vestibulocerebellar tracts deliver real-time information about body position, movement, and head orientation, while corticopontine projections convey top-down signals reflecting motor plans, attentional set, and emotional tone. Within the cerebellar cortex and deep nuclei, these inputs are temporally and spatially aligned to predict error and adjust gain accordingly. The cerebellum influences output through projections to motor, prefrontal, limbic, and autonomic control centers via the thalamus and brainstem. When functioning normally, this allows for precise scaling of muscle tone, coordination, thought, emotion, and visceral responses. When cerebellar input or modulation is impaired, the result is dysmetric motor output, disrupted cognitive timing, emotional instability, and dysregulated autonomic or neuroendocrine states.

Following concussion, injury to midline structures, often located at the interface of tethered and mobile brain tissue, compromises their ability to regulate reflex circuits. The result is a loss of state-dependent gating, which permits primitive and postural reflexes to re-emerge or become exaggerated (Taylor and Gandevia, 2004; Blythe, 2005). These reflexes then contribute aberrant afferent input and maladaptive motor output, forming a feedback loop that destabilizes large-scale networks and reduces their adaptive capacity.

This feedback loop is bidirectional. Reflex disinhibition not only reflects impaired suprasegmental control but also generates maladaptive bottom-up input that interferes with cortical processing. For example, distorted proprioceptive signals from cervical afferents, shaped by retained or dysregulated reflexes, can alter spatial orientation, gaze stability, and postural tone, placing increased demand on already compromised cortical systems (Kristjansson and Treleaven, 2009; Pettorossi and Schieppati, 2014). Disruption of cerebellar modulation of the VOR or VSR can result in inaccurate eye movements and unstable posture, further distorting re-afferent sensory input. Similarly, dysregulation of reticular gain settings can create chronically elevated or unstable muscle tone, entraining maladaptive sensorimotor feedback loops. This afferent noise disrupts predictive coding and sensorimotor weighting, contributing to inefficient cognitive and motor behavior. As these maladaptive patterns persist, they constrain the brain’s capacity for network-level reorganization, decrease cognitive reserve, and diminish neural efficiency.

Functional imaging in chronic mTBI consistently reveals reduced network modularity, increased local clustering, and impaired transitions between major networks (Hillary et al., 2014; Wang et al., 2018). These signatures reflect not only diffuse axonal injury, but also ongoing subcortical dysregulation rooted in reflex circuit instability. Reflex circuit disruption is not merely a secondary effect of brain injury, it is a functional engine capable of anchoring and perpetuating dysfunction across cortical and subcortical systems. Recognizing these circuits as active participants in network degradation reframes them as central to the persistence and heterogeneity of post-concussive symptoms. Early identification and modulation of reflex dysfunction may offer a path to restoring network adaptability and achieving meaningful clinical recovery.

## Reflex-mediated disruption of thalamocortical function and network coordination

The thalamus operates as the brain’s state-dependent gain modulator, integrating and synchronizing sensory inputs, cognitive signals, and motor plans across cortico-cortical and subcortical circuits. Reflex circuits, especially those involving proprioceptive, vestibular, and postural inputs, are critically shaped by thalamic relay nuclei, including the ventral posterolateral (VPL), ventral lateral (VL), and mediodorsal (MD) nuclei (L. Zhang et al., 2004; J. Zhang et al., 2016). These nuclei support sensorimotor integration, attentional salience, executive modulation, and many other executive functional networks. Disruption in reflex circuits can therefore directly alter thalamocortical dynamics.

- Key Point: Sensory input contributes to basal ganglia gain modulation by providing real-time feedback about the internal and external environment. Signals, particularly vestibular, proprioceptive, somatosensory, and visual, are first processed by primary and associative cortices, which filter for relevance and context before informing prefrontal, motor, and limbic regions. These top-down areas generate predictive models and goals, then project to the striatum where they converge with bottom-up sensory input. Dopaminergic modulation within the striatum adjusts the salience and timing of this integration, influencing the balance of direct, indirect, and hyperdirect pathways. This determines inhibitory output from the globus pallidus internus and substantia nigra pars reticulata to the thalamus, modulating the gain of thalamocortical signaling. When intact, this system scales motor, cognitive, affective, and limbic output to match internal state and environmental demands. When disrupted, it produces abnormal gain states, manifesting as bradykinesia or hyperkinesia, bradyphrenia or hyperphrenia, and dysregulated mood or motivation.

After concussion, dysregulated afferent input from reflex circuits, especially vestibular signaling, creates excessive or unbalanced demands on thalamic processing. These distorted signals are not simply misrouted; they are inappropriately weighted by the thalamus, which begins to prioritize aberrant input, overwhelming higher-order circuits with unreliable or maladaptive information (Kristjansson and Treleaven, 2009; Pettorossi and Schieppati, 2014). This misprioritization impairs thalamocortical synchronization, leading to degraded signal-to-noise ratios in motor planning, attentional networks, and executive systems. Moreover, the cerebellum’s disruption compounds thalamic disintegration. The cerebellar deep nuclei, via the superior cerebellar peduncle, project heavily to VL and MD thalamic nuclei. Impaired cerebellar output from damaged peduncles or deep nuclei alters the thalamus’ predictive coding capabilities, its ability to model and filter incoming information. Without coherent cerebellar input, the thalamus struggles to modulate sensory precision, further destabilizing sensorimotor gating and attentional control (Middleton and Strick, 2001; Sokolov et al., 2017). The cumulative effect is rigid, inefficient network coordination. The anterior cerebellar network, default mode network (DMN), salience network (SN), and frontoparietal network (FPN), which depend on thalamic gating to maintain task flexibility, become dysregulated. This manifests clinically as slowed processing, executive fatigue, and cognitive overload, especially during dual-task or novel situations (Hillary et al., 2014; Wang et al., 2018; Zhou et al., 2012; Middleton and Strick, 2001; Sokolov, Miall, and Ivry 2017). In this framework, reflex impairment is not a peripheral symptom but a core mechanism driving thalamocortical dysfunction and cortical network rigidity. Targeting the reflex systems that distort thalamic input, whether through sensorimotor retraining, vestibular recalibration, or cervical integration, may unlock more effective recovery by restoring the foundational coordination between subcortical reflex modulation and cortical computation.

## The network entrapment by reflex dysfunction (NERD) model

To integrate the preceding anatomical, physiological, and systems-level insights into a unified explanatory framework, we propose the Network Entrapment by Reflex Dysfunction (NERD) Model. This model posits that persistent post-concussive symptoms arise from a self-perpetuating cycle in which impaired reflex circuits, whether structurally damaged or functionally disinhibited, produce aberrant motor output that generates distorted sensory feedback. This maladaptive input destabilizes thalamocortical coordination and constrains large-scale brain network flexibility.

At its core, the NERD model conceptualizes reflex dysfunction not as an epiphenomenon but as a primary mechanism of network entrapment. Following concussion, injury to integrative nodes such as the brainstem, cerebellum, thalamus, and sensory integration areas of the cortex disrupts both reflex gain modulation and top-down gating mechanisms. This breakdown permits the persistence or re-emergence of primitive and postural reflexes, introducing maladaptive sensorimotor patterns into cortical processing systems. Compounding this, cortical injury independently impairs sensory integration and predictive filtering, further amplifying distorted afferent signaling. The result is a recursive feedback loop: dysregulated reflexes create aberrant motor responses, which generate maladaptive sensory input that misguides thalamocortical and cortical processing. This overloads higher-order systems, reduces inhibitory control, and reinforces subcortical dysfunction. In an effort to maintain stability, the brain recruits secondary reflexes and develops compensatory behaviors that, over time, become maladaptive and ingrained through negative plasticity. This process entrains rigid activity patterns across the default mode, salience, and sensorimotor networks, manifesting as cognitive inflexibility, postural instability, autonomic dysregulation, and affective disturbances. By framing post-concussive dysfunction as an emergent property of reflex-mediated network entrapment, the NERD model bridges clinical observations with systems neuroscience. It offers a mechanistic rationale for targeted, bottom-up rehabilitation and lays the foundation for formal mathematical modeling, neurophysiological testing, and outcome-driven clinical research.

## Integration of cortical injury within the reflex-entrapment framework

Although the NERD model emphasizes reflex circuit dysfunction as a diaschitic mechanism of persistent post-concussive symptoms, it does not preclude the contributory role of direct cortical injury. Structural or functional damage to frontal, prefrontal, temporal, parietal, or occipital cortical regions, particularly areas involved in executive control, spatial awareness, vision, and motor initiation, can result in a myriad of signs and symptoms. Such deficits reflect diminished capacity for predictive filtering, sensory prioritization, and adaptive behavioral modulation. Importantly, cortical dysfunction may exacerbate the impact of aberrant afferent input originating from dysregulated reflex circuits. In this context, impaired bottom-up signaling and compromised top-down processing interact synergistically, producing a recursive loop in which degraded sensory input is insufficiently filtered, integrated, or inhibited, further amplifying maladaptive network activity. Rather than representing distinct or competing etiologies, cortical and subcortical injuries are best understood as interdependent contributors to network rigidity and the heterogeneous clinical manifestations of chronic concussion. This interdependence suggests that interventions targeting foundational sensorimotor mechanisms may have upstream effects on cortical function, an idea explored in subsequent sections.

## Constrained adaptation and the limits of stability: anchoring forces, psychological defaults, and the tipping point in the NERD model

The NERD model posits that following neurological insult, whether due to concussion, chronic stress, or reflex disinhibition, the nervous system does not descend into chaos. Instead, it reorganizes into new, often less efficient but stable configurations. This process, referred to as constrained adaptation, reflects the nervous system’s deep reliance on anchoring rhythms, hardwired scaffolds, and psychological defaults that collectively prevent catastrophic breakdown. The system does not spiral aimlessly; rather it recalibrates toward a new, lower-efficiency set point that prioritizes coherence over optimality. This aligns closely with established biological models of homeostasis and allostasis. While homeostasis maintains stability through fixed set points, allostasis allows the system to shift its set points under sustained load (Sterling, 2012; McEwen and Wingfield, 2003). The NERD model extends this framework to explain how the nervous system absorbs and reorganizes around post-injury perturbation without disintegration provided the compensatory capacity is not overwhelmed.

## Anchors of constrained adaptation: environmental, structural, and psychological

Three primary classes of stabilizing input maintain coherence in the post-injury nervous system:

### Predictable environmental rhythms

The brain is embedded in a world of structured, rhythmic input. These include the omnipresent vector of gravity and light–dark cycles.

- Gravity provides continuous directional feedback to the vestibular and proprioceptive systems, shaping axial tone and orientation (Angelaki and Cullen, 2008).
- Circadian cycles entrain hormonal, metabolic, and attentional rhythms through the suprachiasmatic nucleus (Czeisler et al., 1999).

These exogenous rhythms serve as external constraint vectors, acting as passive scaffolds around which the brain can rebuild organization, even when intrinsic coordination is disrupted.

### Endogenous structural scaffolds

Internally, the nervous system contains primitive but persistent structures that generate patterned outputs with or without cortical involvement.

- Central Pattern Generators (CPGs) in the spinal cord and brainstem produce rhythmic motor sequences (e.g., gait, respiration) independent of sensory input (Grillner and El Manira, 2020).
- Primitive and postural reflexes, when released from cortical inhibition, provide fallback coordination templates based on pre-programmed motor strategies (Zafeiriou, 2004).
- Respiratory and cardiac rhythms introduce recurring afferent perturbations that entrain neural oscillations, autonomic tone, and interoceptive awareness (Zaccaro et al., 2018).

These structures are not high-functioning, but they are robust. When higher-order regulation fails, they provide a default operational layer that favors pattern over precision.

### Psychological defaults

In parallel, the mind possesses self-preserving heuristics that act to preserve identity, agency, and coherence.

- Defensive strategies such as withdrawal, denial, or emotional flattening serve to reduce overload and preserve internal stability (Vaillant, 1992).
- The brain favors predictive stability, even when distorted, over interpretive chaos. This includes narrative construction and cognitive minimization of threat, helping maintain a coherent sense of self (Friston, 2010).
- Behavioral withdrawal, social disengagement, or energy conservation reflect a psychological analogue to metabolic downscaling, a hallmark of allostatic overload (Porges, 2009).

The psychological defaults operate like low-variability cognitive CPGs that are habitual, protective, and often difficult to disrupt. While not inherently pathological, they become maladaptive when they inhibit reintegration. Together, the external rhythms, internal circuits, and psychological programs act as a multi-layered constraint system that ensures stability under load. The nervous system’s capacity to reorganize within these constraints is shaped by neural reserve, the efficiency, redundancy, and flexibility of the individual’s neural architecture (Stern, 2002). The following relationship captures the NERD model’s foundation and explains why many patients settle into stable but dysfunctional attractors, they have not “spiraled out” but instead become entrapped in a low-flexibility state that the nervous system perceives as survivable:


$$\begin{array}{l} \text{Constrained Adaptation}=\text{Allostatic Buffering}+\text{Environmental}\;\\ \text{Anchors}+\text{Neural Reserve}+\\ \text{Psychological Defaults}. \end{array}$$

## When constrained adaptation fails: rigidity and fragmentation

Constrained adaptation is not unlimited. When the total burden (whether reflexive, metabolic, vestibular, emotional, or perceptual) exceeds the system’s allostatic capacity, stabilization becomes impossible. At this point, the system crosses a critical threshold beyond which functional reorganization cannot be maintained.

This does not result in chaos per se, but in decompensation through one of two patterns:

1. Rigid Attractor Dominance: The system becomes locked into narrow, inflexible patterns that resist change. This may manifest as fixed postural asymmetry, hypervigilance, persistent threat monitoring, or chronic sympathetic overactivation. These states are energetically expensive yet reflexively stable, like over-fortified positions that are difficult to abandon (Turrigiano, 2012).
2. Fragmentation and Network Instability: Alternatively, the system may fluctuate between competing attractors, never achieving stable integration. This is seen in episodic dizziness, fluctuating symptoms, affective lability, or inconsistent motor control reflecting an unstable attractor landscape (Sharp et al., 2014; Friston, 2010).

This threshold can be conceptualized as a point where entrapment outweighs the system’s total buffering capacity, leading to the failure of constrained adaptation and eventual decompensation - either into rigidity or fragmentation. In practice, this means not all patients can self-recover. When anchoring mechanisms and neural reserve are overwhelmed, external intervention is required to restore variability, resolve asymmetry, and scaffold reorganization. This is the therapeutic aim of the NERD model: to reintroduce adaptive flexibility and disrupt pathological attractors through structured, multimodal rehabilitation.

While the NERD model has thus far been presented through anatomical, physiological, and systems-level reasoning, its recursive structure also lends itself to formal modeling. To represent the dynamic interactions between reflex circuit injury, gain degradation, behavioral output, and cortical adaptability, we propose a differential equation framework. This mathematical formalization is not a predictive model per se, but a structured scaffold for quantifying and simulating the model’s internal logic. It offers a mechanism for translating clinical intuition and systems neuroscience into testable, time-evolving dynamics.

## Mathematical formalization of the NERD model

The Network Entrapment by Reflex Dysfunction (NERD) model can be expressed as a differential equation that formalizes its cascading, recursive dynamics. This formulation captures how reflex circuit injury leads to aberrant motor output, distorted sensory feedback, degraded gain modulation, and maladaptive behavioral patterns - ultimately driving cortical rigidity. It provides a dynamic systems structure that aligns with clinical observations and lays a foundation for future computational modeling.

The anatomical substrates, reflex pathways, and modulatory systems described here are well established in both classical and contemporary neurology. The roles of subcortical reflex circuits, thalamocortical loops, and cerebellar and basal ganglia modulation in motor, cognitive, and autonomic control have been thoroughly documented in experimental and clinical neuroscience. What distinguishes this framework is not the novelty of its anatomical claims, but the integration of mathematical modeling and systems dynamics to examine how these circuits interact over time. By treating neurological function as a dynamic system governed by identifiable domains, gain states, and degradation pathways, the NERD model bridges traditional neuroanatomy with principles from control theory and computational neuroscience.

The proposed formalism is presented in Equation 1, with corresponding symbol definitions provided in Table 3.

**Table 3:** Symbol glossary.

Symbol	Description	Role in Model
I(t)	Reflex circuit injury (brainstem, midbrain)	Primary injury input; initiates reflex dysfunction
C_I_(t)	Cortical injury load (sensory integration + reflex gating areas)	Secondary injury input; disrupts sensory processing and gain modulation
α (alpha)	Reflex vulnerability coefficient	Scales how strongly injury affects reflexes
γ (gamma)	Reflex injury to motor output gain	Converts reflex dysfunction into aberrant motor output
μ (mu)	Motor output to sensory feedback distortion gain	Converts motor abnormalities into distorted afferent signals
σ (sigma)	Cortical injury contribution to sensory feedback distortion	Adds sensory noise from impaired integration
λ (lambda)	Sensory distortion to gain modulation disruption factor	Converts distorted feedback into degraded gain control
ξ (xi)	Cortical injury’s direct disruption of gain modulation	Further reduces gain modulation from cortical injury
G₀	Baseline gain modulation capacity	The ideal/reference value for gain control
ρ (rho)	Reflex recruitment sensitivity to gain modulation loss	Determines how quickly primitive reflexes re-emerge
ν (nu)	Reflex contribution to maladaptive behavior	Weight of secondary reflexes in shaping output
κ (kappa)	Aberrant motor contribution to maladaptive behavior	Weight of motor dysfunction in behavioral shaping
χ (chi)	Behavioral impact on network degradation	Converts behavior into cortical dysfunction
ψ (psi)	Gain modulation impact on network degradation	Converts gating dysfunction into cortical dysfunction
η (eta)	Global network degradation rate	Scales how quickly cortical adaptability declines
N(t)	Cortical network adaptability	Output variable; reflects brain network flexibility or entrapment

The symbol glossary organizes every variable and parameter that appears in the NERD equation into functional strata: (i) Injury drivers. I(t) represents the axonal injury that creates immediate insult to reflex-circuitry in the subcortical structures, whereas C_I_(t) captures the primary cortical injury (if present) or resultant secondary cortical burden that compromises sensory-motor integration, cognitive processing and reflex gating. Combined they constitute the traumatic event that sets the cascade in motion. (ii) Link-specific gains. The coefficients α, γ, μ, σ, λ, and ξ form the mechanistic bridges that translate raw injury into network perturbation. α modulates the sensitivity to reflex circuits to injury. γ represents scale reflex dysfunction into the motor domain. μ and σ represent reflex-derived and cortex-derived noise, respectively, into afferent sensory streams. λ channels that sensory noise into loss of gain control, and ξ models direct cortical excision of gating capacity. Collectively, these parameters describe how anatomical disruption of reflex circuits, normally simple sensorimotor integrations, produces aberrant motor output and distorted sensory input. This altered information re-enters the system, degrading gain modulation and destabilizing both local integration centers and large-scale networks. The result is compromised perception, affect, cognition, and motor function, as reflected in the progressive decline of N(t). (iii) Reference reserve. G_0_ represents the idealized dynamic range for gain modulation, a benchmark against which all functional degradation is measured. is measured. (iv) Behavioral weighting factors. ρ,ν, and κ determine how disinhibited reflex activity and aberrant motor output are expressed as overt behavior. These terms comprise the “behavioral-load” block of the equation, capturing how bottom-up dysfunction is enacted. (v) Degradation translators. χ and ψ convert behavioral chaos and gain-control failure, respectively, into cortical degradation rates. These coefficients serve as local levers through which interventions, whether rehabilitative or pharmacologic, can act. (vi) Global tempo. η acts as a master time constant, globally scaling both degradation pathways; a higher η accelerates entrapment even if individual gains are modest. (vii) System output. N(t) is the model’s sole state variable and outcome measure. Its value reflects the moment-to-moment adaptability, or conversely, the entrapment, of the cortical network.


$$\frac{\mathrm{dN}}{\mathrm{dt}}=-\eta (\begin{array}{l} X[\text{kγαI}(t)+\nu \rho (\text{λμγαI}(t)+\sigma C_{I}(t))]+\\ \psi [G_{0}-\text{λμγαI}(t)-\lambda \sigma C_{I}(t)-\xi C_{I}(t)] \end{array})$$ (1)

Equation 1: The NERD Equation. Every millisecond of distorted sensorimotor traffic and each notch of lost gain control combine, at rates χ and *ψ*, globally scaled by *η*, to entrap the cortical network in progressively rigid dynamics.

For clarity of exposition we present the core NERD equation with receptor inflow subsumed in G_0_. However, the model is readily extended by replacing G_0_ with an extension to Equation 1 defined as:


$$H(t)=\max\{0,G_{0}+\zeta R_{\mathrm{net}}(t)\},R_{\mathrm{net}}(t)=R_{0}+R_{\text{stim}}(t)+R_{\text{loss}}(t)$$ (2)

Equation 2 extension. H(t) - Receptor-Driven Gain Support. The term H(t) represents afferent input from receptors that stabilizes reflex gain. It supports the baseline gain reserve G_0_ and models how sensory stimulation, whether preserved or impaired, influences system resilience and responsiveness to rehabilitation.

Describing the spare dynamic range that the brain’s gating circuitry still has in reserve that lets it turn sensory and motor gains *up* or *down* without running into hard limits or noise saturation.

The right-hand side of the NERD differential equation comprises two dynamically intertwined drivers of decompensation (Behavioral load + Gain-control collapse), each multiplied by its own local gain (χ or ψ) and globally scaled by the time constant η. The two component drivers are shown below in Equation 3.


$$\frac{\mathrm{dN}}{\mathrm{dt}}=-\eta (\underset{\text{Behavioural}\_\text{load}}{\underset{︸}{X[\text{kγαI}(t)+\nu \rho (\begin{array}{l} \text{λμγαI}(t)+\\ \sigma C_{I}(t) \end{array})]}}+\underset{\text{Gain}-\text{control collapse}}{\underset{︸}{\psi [\begin{array}{l} G_{0}-\text{λμγαI}(t)-\\ \lambda \sigma C_{I}(t)-\xi C_{I}(t) \end{array}]}})$$ (3)

Equation 3 decomposition: Decomposition of the NERD equation. The NERD equation separates into two interacting components; behavioral load (χ-weighted) and gain control collapse (ψ-weighted), which jointly drive the decline in cortical adaptability N(t).

The behavioral load represents the energetic and structural stress that overt reflex-mediated behavior places on cortical circuits. Reflex-circuit injury I(t) amplifies outgoing motor commands through the gain *γα*, while distorted reafference and cortical noise (*λμ*γ*α*I+*σC_I_*) destabilise all reflexes governed by gain modulation. These dysregulated loops, weighted by ρ and expressed in behavior with weight *ν*, combined with exaggerated motor drive (*κ*γαI). The coefficient χ transforms the resulting blend of excessive metabolic demand, electrophysiological hyper-synchrony, and micro-structural shear into a rate at which cortical adaptability erodes.

The gain-control collapse measures the shortfall between the ideal gating reserve G_0_ and the degraded capacity available in real time. Aberrant motor output feeds proprioceptive noise back into the system (λμγαI); cortical injury generates intrinsic sensory noise (λσ*C_I_*); and direct damage to gating circuits (*ξC_I_*) removes functional tissue altogether. The wider this gap grows, the less freedom the network retains to tune reflex gains, posture, eye movements, and other sensorimotor loops. The coefficient ψ converts this dwindling head-room into a concrete degradation rate, pushing the cortex toward progressively rigid, low-dimensional dynamics.

These two processes, behavioral chaos funnelled through χ and gating failure funnelled through ψ, operate under the global clock set by η. Cortical network adaptability N(t) therefore declines whenever either driver outweighs the system’s remaining plastic reserve, ultimately entrapping the brain in progressively rigid and maladaptive network dynamics (Table 2).

### Parameter interpretation

All parameters in the NERD equations are dimensionless scaling factors (0 ≤ parameter ≤ 1) representing relative influence weights within the system. They are not directly measurable physical quantities but rather theoretical constructs that capture the strength of interactions between model components (Wilson and Cowan, 1972; Breakspear, 2017). This parameterization approach is standard in computational neuroscience modeling, where the goal is to capture system dynamics at a level of abstraction that balances mechanistic detail with empirical tractability (Dayan and Abbott, 2001). The specific values for these parameters will be estimated from empirical data using Bayesian inference methods during validation (see Protocol 4), with the ranges provided in Table 4 serving as informed priors based on existing literature.

**Table 4:** NERD model parameter justification overview with supporting literature.

Parameter	Symbol	Mechanistic role	Physiological basis	Key supporting literature	Estimated range
Reflex-to-motor scaling	α (alpha)	Converts reflex dysfunction into aberrant motor output	Vestibulospinal and reticulospinal tract gain; primitive reflex disinhibition following cortical injury	Fife and Baloh (1993); Guskiewicz (2011); Mucha et al. (2022); Pearce (2008)	0.3–0.8
Reflex-to-motor conversion	γ (gamma)	Transforms reflex circuit injury into motor control errors	Brainstem reflex pathway integrity; tonic neck reflex and vestibulo-ocular reflex (VOR) gain modulation	Alsalaheen et al. (2010); Fino et al. (2018); Hoffer et al. (2009)	0.4–0.9
Motor-to-sensory gain	μ (mu)	Motor output distorts sensory feedback (efference copy mismatch)	Cerebellar forward model prediction error; sensory reweighting in vestibular disorders	Angelaki and Cullen (2008); Cullen (2012); Horak (2006); Peterka (2002)	0.2–0.6
Sensory-to-gain modulation	λ (lambda)	Distorted sensory input degrades thalamocortical gain control	Thalamic gating dysfunction; pulvinar and mediodorsal nuclei in sensory integration	Halassa and Kastner (2017); Hwang et al. (2017); Sherman (2016)	0.3–0.7
Gain-to-reflex sensitivity	ρ (rho)	Loss of gain modulation increases primitive reflex recruitment	Cortical disinhibition of brainstem reflexes; reduced prefrontal-subcortical connectivity	Caeyenberghs et al. (2012); Hillary et al. (2014); Sharp et al. (2014)	0.4–0.8
Reflex-to-behavior weight	ν (nu)	Secondary reflexes contribute to maladaptive behavioral patterns	Postural instability, gaze dysfunction, and autonomic dysregulation in mTBI	Hoffer et al. (2004); Kleffelgaard et al. (2012); Leddy et al. (2016)	0.2–0.5
Behavior-to-network impact	χ (chi)	Maladaptive behavior accelerates cortical network degradation	Activity-induced metabolic stress; neuroinflammation from sustained dysfunction	Barkhoudarian et al. (2011); Johnson et al. (2013); Xiong et al. (2013)	0.3–0.6
Motor-to-behavior weight	κ (kappa)	Aberrant motor output shapes maladaptive behavioral strategies	Compensatory movement patterns; fear-avoidance behavior in chronic symptoms	Hou et al. (2012); Schneider et al. (2014); Terry and Miller (2015)	0.2–0.5
Cortical injury-to-sensory	σ (sigma)	Cortical injury adds sensory integration noise	Impaired multisensory integration; disrupted sensory cortex connectivity	Mayer et al. (2011); Slobounov et al. (2010); Zhou et al. (2012)	0.3–0.7
Cortical injury-to-gain	ξ (xi)	Direct cortical injury disrupts gain modulation capacity	Prefrontal and parietal injury impairs top-down attentional control	Bigler (2013); Kinnunen et al. (2011); Scheibel (2017)	0.4–0.8
Gain-to-network degradation	ψ (psi)	Gating dysfunction accelerates network rigidity	Thalamocortical dysrhythmia; reduced default mode network flexibility	Bonnelle et al. (2011); Llinás et al. (1999); Stevens et al. (2012)	0.3–0.7
Adaptive constraint	η (eta)	Limits maximum network degradation (homeostatic reserve)	Neuroplasticity capacity; cognitive reserve; compensatory recruitment	Bigler (2001); Stern (2002); Strangman et al. (2005)	0.1–0.3

## Parameter justification and literature support

### Nature and interpretation of model parameters

The NERD model parameters (α, *γ*, *μ*, *λ*, ρ, ν, χ, κ, σ, ξ, ψ, η) are theoretical, non-dimensional scaling factors constrained between 0 and 1. These parameters represent relative influence weights within the system rather than directly measurable physical quantities with units (Wilson and Cowan, 1972; Dayan and Abbott, 2001). This approach is standard in computational neuroscience, where dimensionless parameters capture the strength of interactions between model components without requiring direct one-to-one correspondence with observable physiological variables (Doya, 2007; Breakspear, 2017).

The ranges provided in Table 4 are informed experimental priors derived from published literature on reflex function, network dynamics, gain modulation, and clinical observations. These priors serve as starting points for Bayesian parameter estimation during empirical validation, as detailed in Protocol 4 (Friston, 2010; Gelman et al., 2013). As empirical data accumulates from reflex assessments, neuroimaging, and clinical outcomes, posterior distributions will refine these estimates, with uncertainty quantified through credible intervals. This iterative refinement process allows the model to be progressively calibrated against real-world data while maintaining theoretical coherence (Stephan et al., 2017).

It is important to emphasize that these parameters are not fixed values but rather represent our current best estimates based on available evidence. The model’s validity does not depend on the precise accuracy of these initial values, but rather on whether the overall framework—with parameters estimated from data—can predict clinical outcomes, recovery trajectories, and intervention responses better than alternative models (Daunizeau et al., 2014). The explicit Bayesian approach to parameter estimation ensures that uncertainty is appropriately quantified and that the model remains falsifiable through empirical testing.

The twelve parameters (α, β, γ, δ, λ, μ, *ν*, ρ, σ, ξ, χ, ψ, κ) of the NERD model govern the dynamics of network degradation following reflex circuit injury. Each parameter represents a specific mechanistic relationship between functional nodes, grounded in empirical observations from neuroscience, clinical neurology, and systems biology. This section provides explicit justification for each parameter (Table 4), linking model assumptions to established literature.

### Detailed parameter justifications

#### α (Alpha): reflex-to-motor scaling factor

Mechanistic Role: Determines the magnitude by which reflex circuit dysfunction translates into aberrant motor output.

Physiological Basis: - Vestibulospinal tract (VST) and reticulospinal tract (RST) provide direct pathways from brainstem reflex centers to spinal motor neurons (Fife and Baloh, 1993). - Following TBI, disinhibition of primitive reflexes (tonic neck reflex, asymmetric tonic neck reflex) increases motor output variability (Pearce, 2008). - VOR gain abnormalities correlate with postural instability and gait dysfunction in mTBI patients (Mucha et al., 2022).

Supporting Evidence: - Guskiewicz (2011) demonstrated that balance deficits persist in 30% of concussed athletes beyond clinical recovery, suggesting sustained reflex dysfunction. - Hoffer et al. (2009) showed that VOR abnormalities predict chronic symptom severity.

Estimated Range: 0.3–0.8 (higher values indicate greater reflex-to-motor conversion efficiency).

#### γ (Gamma): reflex-to-motor conversion efficiency

Mechanistic Role: Converts reflex circuit injury into motor control errors.

Physiological Basis: - Brainstem reflex pathways (vestibular nuclei, reticular formation) are vulnerable to rotational acceleration forces (Fino et al., 2018). - VOR and cervico-ocular reflex (COR) disruption leads to oculomotor dysfunction (Alsalaheen et al., 2010).

Supporting Evidence: - Fino et al. (2018) found that VOR gain asymmetry persists in 40% of mTBI patients at 6 months post-injury. - Alsalaheen et al. (2010) reported that vestibular dysfunction is present in 50–80% of acute mTBI cases.

Estimated Range: 0.4–0.9.

#### μ (Mu): motor-to-sensory feedback distortion

Mechanistic Role: Aberrant motor output distorts sensory feedback through efference copy mismatch.

Physiological Basis: - The cerebellum generates forward models that predict sensory consequences of motor commands (Cullen, 2012). - Mismatch between predicted and actual sensory feedback indicates motor error (Angelaki and Cullen, 2008). - In vestibular disorders, sensory reweighting shifts reliance from vestibular to visual or proprioceptive cues (Peterka, 2002; Horak, 2006).

Supporting Evidence: - Cullen (2012) demonstrated that cerebellar injury impairs sensory prediction, leading to increased reliance on reactive control. - Horak (2006) showed that sensory reweighting failures contribute to chronic balance dysfunction.

Estimated Range: 0.2–0.6.

#### Λ (Lambda): sensory-to-gain modulation disruption

Mechanistic Role: Distorted sensory input degrades thalamocortical gain control.

Physiological Basis: - The thalamus (pulvinar, mediodorsal nuclei) gates sensory information to cortex (Sherman, 2016). - Thalamic relay neurons modulate cortical excitability via gain control mechanisms (Halassa and Kastner, 2017). - TBI disrupts thalamocortical connectivity, impairing attentional gating (Hwang et al., 2017).

Supporting Evidence: - Hwang et al. (2017) found reduced thalamic functional connectivity in chronic mTBI patients with persistent symptoms. - Halassa and Kastner (2017) demonstrated that thalamic dysfunction impairs cortical signal-to-noise ratio.

Estimated Range: 0.3–0.7.

#### ρ (Rho): gain-to-reflex recruitment sensitivity

Mechanistic Role: Loss of gain modulation increases primitive reflex recruitment.

Physiological Basis: - Cortical inhibition suppresses primitive reflexes during development (Pearce, 2008). - TBI reduces prefrontal-subcortical connectivity, disinhibiting brainstem reflexes (Hillary et al., 2014). - Reduced cortical control allows re-emergence of tonic neck reflexes and grasp reflexes (Sharp et al., 2014).

Supporting Evidence: - Caeyenberghs et al. (2012) showed reduced white matter integrity in corticospinal tracts correlates with motor dysfunction. - Hillary et al. (2014) demonstrated that reduced prefrontal connectivity predicts chronic symptom burden.

Estimated Range: 0.4–0.8.

#### ν (Nu): reflex-to-behavior contribution weight

Mechanistic Role: Secondary reflexes contribute to maladaptive behavioral patterns.

Physiological Basis: - Postural instability from vestibular dysfunction leads to compensatory movement strategies (Kleffelgaard et al., 2012). - Autonomic dysregulation (increased sympathetic tone) from brainstem injury affects exercise tolerance (Leddy et al., 2016).

Supporting Evidence: - Hoffer et al. (2004) found that vestibular rehabilitation reduces symptom severity in 80% of mTBI patients. - Leddy et al. (2016) showed that autonomic dysfunction predicts exercise intolerance.

Estimated Range: 0.2–0.5.

#### χ (Chi): behavior-to-network degradation impact

Mechanistic Role: Maladaptive behavior accelerates cortical network degradation.

Physiological Basis: - Sustained neuroinflammation from chronic dysfunction impairs neuroplasticity (Barkhoudarian et al., 2011). - Activity-induced metabolic stress exacerbates mitochondrial dysfunction (Xiong et al., 2013). - Chronic symptoms activate microglia, perpetuating neurodegenerative cascades (Johnson et al., 2013).

Supporting Evidence: - Barkhoudarian et al. (2011) demonstrated that chronic neuroinflammation persists years after mTBI. - Xiong et al. (2013) showed that mitochondrial dysfunction correlates with symptom severity.

Estimated Range: 0.3–0.6.

#### *κ* (Kappa): motor-to-behavior feedback weight

Mechanistic Role: Aberrant motor output shapes maladaptive behavioral strategies.

Physiological Basis: - Compensatory movement patterns (e.g., stiff gait, reduced head movement) reduce sensory input variability (Hou et al., 2012). - Fear-avoidance behavior in chronic pain and post-concussion syndrome perpetuates dysfunction (Schneider et al., 2014).

Supporting Evidence: - Terry and Miller (2015) found that kinesiophobia (fear of movement) predicts chronic post-concussion symptoms. - Schneider et al. (2014) showed that graded exercise therapy reduces fear-avoidance and improves outcomes.

Estimated Range: 0.2–0.5.

#### *σ* (Sigma): cortical injury-to-sensory noise

Mechanistic Role: Cortical injury adds sensory integration noise.

Physiological Basis: - Impaired multisensory integration in parietal and temporal cortices increases sensory conflict (Mayer et al., 2011). - Disrupted default mode network (DMN) connectivity impairs internal model updating (Zhou et al., 2012).

Supporting Evidence: - Slobounov et al. (2010) found that sensory integration deficits persist beyond clinical recovery. - Mayer et al. (2011) demonstrated that DMN disruption correlates with cognitive symptoms.

Estimated Range: 0.3–0.7.

#### *ξ* (Xi): cortical injury-to-gain modulation loss

Mechanistic Role: Direct cortical injury disrupts gain modulation capacity.

Physiological Basis: - Prefrontal cortex (PFC) provides top-down attentional control to thalamus (Bigler, 2013). - Parietal cortex injury impairs spatial attention and sensory weighting (Scheibel, 2017). - Reduced PFC-thalamic connectivity impairs cognitive flexibility (Kinnunen et al., 2011).

Supporting Evidence: - Bigler (2013) showed that PFC volume loss correlates with executive dysfunction. - Kinnunen et al. (2011) demonstrated that reduced PFC connectivity predicts chronic symptoms.

Estimated Range: 0.4–0.8.

#### *ψ* (Psi): gain-to-network degradation amplification

Mechanistic Role: Gating dysfunction accelerates network rigidity.

Physiological Basis: - Thalamocortical dysrhythmia (TCD) generates abnormal oscillations that impair cortical processing (Llinás et al., 1999). - Reduced DMN flexibility limits adaptive network reconfiguration (Bonnelle et al., 2011).

Supporting Evidence: - Llinás et al. (1999) proposed TCD as a mechanism for chronic neurological symptoms. - Stevens et al. (2012) found reduced DMN connectivity in chronic mTBI patients.

Estimated Range: 0.3–0.7.

#### *η* (Eta): adaptive constraint (homeostatic reserve)

Mechanistic Role: Limits maximum network degradation through compensatory mechanisms.

Physiological Basis: - Cognitive reserve (education, premorbid intelligence) buffers against injury effects (Stern, 2002). - Neuroplasticity allows functional reorganization and compensatory recruitment (Strangman et al., 2005). - Brain volume and white matter integrity predict recovery capacity (Bigler, 2001).

Supporting Evidence: - Stern (2002) demonstrated that higher cognitive reserve predicts better TBI outcomes. - Strangman et al. (2005) showed that compensatory activation patterns emerge during recovery.

Estimated Range: 0.1–0.3 (lower values indicate greater homeostatic capacity).

Each parameter in the NERD model is grounded in established neuroscience and clinical evidence. The ranges provided represent plausible physiological values based on empirical observations, though precise quantification will require experimental validation. Future work will involve:

1. Parameter Estimation: Using clinical datasets (symptom severity, balance metrics, neuroimaging) to constrain parameter values via inverse modeling.
2. Sensitivity Analysis: Identifying which parameters most strongly influence model behavior.
3. Validation Studies: Testing model predictions against longitudinal patient data.

This parameter justification framework ensures that the NERD model is not merely a mathematical abstraction, but a physiologically grounded hypothesis that can be empirically tested and refined.

## Validation and testable predictions

### Building evidence for a mechanistically-promising framework

While the NERD framework is grounded in established neuroscience and informed by clinical observations across multiple settings, comprehensive prospective validation remains essential to establish its predictive accuracy and clinical utility. Preliminary empirical work is underway - a randomized controlled trial testing interventions based on these principles has demonstrated initial feasibility and is currently under peer review (Jay et al., n.d.) - yet these early findings require independent replication and expansion. The validation protocols outlined below are designed to systematically test the framework’s core predictions, refine parameter estimates, and build a robust evidence base that can guide clinical translation.

Rather than prerequisites that must be completed before any clinical exploration, these validation steps represent a roadmap for strengthening evidence for an approach that shows mechanistic promise and preliminary empirical support. The protocols are structured to be conducted in parallel or sequentially, depending on available resources, and are designed to yield both confirmatory evidence and opportunities for model refinement. Each protocol includes explicit falsification criteria, ensuring that the framework can be rigorously tested and modified or rejected if predictions are not supported.

The following sections detail four complementary validation approaches: (1) neuroimaging studies to test predicted network dynamics, (2) longitudinal clinical trials to assess recovery trajectories, (3) computational modeling to validate parameter relationships, and (4) experimental interventions to test causal predictions. Together, these protocols provide a comprehensive strategy for advancing the NERD framework from a mechanistically-grounded hypothesis to a validated clinical tool.

### Core testable predictions

#### Prediction 1: reflex dysfunction predicts symptom severity

Hypothesis: Patients with greater reflex circuit dysfunction (measured by VOR gain asymmetry, postural instability, and primitive reflex disinhibition) will exhibit higher symptom severity and longer recovery times.

Quantitative Prediction: - VOR gain asymmetry >20% → 3-fold increased risk of persistent symptoms at 3 months - Abnormal Sensory Organization Test (SOT) scores (composite <60) → 2-fold increased symptom burden - Presence of ≥2 primitive reflexes → 4-fold increased risk of chronic symptoms.

Measurable Outcomes: - Post-Concussion Symptom Scale (PCSS) scores / Rivermead Post concussion Symptoms Questionaire - Return-to-activity timeline - Quality of life measures (eg. SF-36 or similar instrument).

#### Prediction 2: network degradation correlates with connectivity metrics

Hypothesis: The model’s network degradation metric N(t) should correlate with neuroimaging measures of brain connectivity, particularly in thalamocortical and default mode networks.

Quantitative Prediction: - N(t) decline should correlate (*r* > 0.6) with: - Reduced DMN functional connectivity (fMRI) - Decreased thalamocortical white matter integrity (DTI fractional anisotropy) - Increased thalamocortical dysrhythmia (EEG coherence measures).

Neuroimaging Targets: - Resting-state fMRI: DMN, salience network, executive control network - DTI: Thalamocortical radiations, corpus callosum, superior longitudinal fasciculus - EEG: Alpha/theta power ratios, coherence patterns.

#### Prediction 3: parameter values predict recovery trajectories

Hypothesis: Individual differences in model parameters (estimated from baseline assessments) should predict recovery trajectories.

Quantitative Prediction: - Patients with high *α* (reflex-to-motor scaling) and low *η* (homeostatic reserve) → slow recovery (>6 months) - Patients with low ρ (gain-to-reflex sensitivity) and high η → fast recovery (<6 weeks) - Parameter-based risk stratification should outperform symptom-only models (AUC > 0.75).

Statistical Approach: - Inverse modeling to estimate patient-specific parameters - Survival analysis (time to recovery) - ROC curve analysis for predictive accuracy.

#### Prediction 4: reflex-targeted interventions improve outcomes

Hypothesis: Interventions targeting reflex circuit dysfunction (vestibular rehabilitation, oculomotor training, cerebellar stimulation) should produce greater symptom reduction than standard care.

Quantitative Prediction: - Reflex-targeted therapy → 40% greater symptom reduction vs. standard care - Improvement should be mediated by changes in reflex metrics (VOR gain, SOT scores) - Effects should persist at 6-month follow-up.

Control Conditions: - Active control: General aerobic exercise (matched for time and effort) - Placebo control: Sham vestibular stimulation - Standard care: Symptom-based management.

#### Prediction 5: temporal dynamics match model simulations

Hypothesis: Longitudinal symptom trajectories should match model-predicted dynamics, including potential non-linear transitions and critical thresholds.

Quantitative Prediction: - Symptom severity should follow exponential decay in recovering patients: S(t) = S₀ . e^(−λt) - Patients exceeding critical threshold [N(t) < 0.3] should exhibit persistent symptoms - Model simulations should predict 70% of variance in observed trajectories.

Longitudinal Design: - Weekly assessments for 12 weeks - Monthly assessments for 6 months - Model fitting using nonlinear mixed-effects models.

### Experimental validation protocols

#### Protocol 1: longitudinal reflex-symptom correlation study

Objective: Test whether reflex dysfunction predicts symptom persistence.

Design: - Study Type: Prospective longitudinal cohort - Sample Size: *n* = 100 mTBI patients (power analysis: 80% power to detect *r* = 0.3, *α* = 0.05) - Duration: 6-month follow-up - Inclusion Criteria: Diagnosed mTBI (ACRM criteria), age 18–55, <14 days post-injury - Exclusion Criteria: Previous TBI, neurological disorders, vestibular pathology.

##### Assessments

*Baseline (within 2 weeks of injury):* - VOR gain (video head impulse test) - Postural stability (Sensory Organization Test) - Primitive reflex battery (tonic neck reflex, asymmetric TNR, Moro reflex) - Symptom severity (PCSS) - Cognitive function (ImPACT battery) - Neuroimaging (fMRI, DTI - subset of *n* = 30).

*Follow-up (1, 3, 6 months):* - Repeat all baseline assessments - Return-to-activity status - Quality of life (SF-36).

Primary Outcome: Correlation between baseline reflex dysfunction and symptom severity at 3 months.

Statistical Analysis: - Multiple regression: Symptom severity ~ VOR gain + SOT score + reflex count + age + sex - Mediation analysis: Test if reflex dysfunction mediates injury-symptom relationship - Survival analysis: Time to symptom resolution as function of reflex metrics.

Expected Results (if model is valid): - Baseline reflex dysfunction predicts 30–40% of variance in 3-month symptom severity - VOR gain asymmetry >20% → 3-fold increased risk of persistent symptoms - Reflex metrics add predictive value beyond standard clinical measures.

#### Protocol 2: neuroimaging validation study

Objective: Test whether predicted connectivity patterns correlate with reflex dysfunction and symptom severity.

Design: - Study Type: Cross-sectional case–control with correlational analysis - Sample Size: *n* = 50 mTBI patients with persistent symptoms (>3 months), *n* = 30 healthy controls - Neuroimaging Modalities: Resting-state fMRI, DTI, EEG.

##### Assessments

*All Participants:* - Resting-state fMRI (10 min, eyes open) - DTI (64 directions, b = 1,000 s/mm^2^) - EEG (5 min resting state, eyes closed and open) - Reflex assessment battery (VOR, SOT, primitive reflexes) - Symptom severity (PCSS).

##### Neuroimaging analysis

*fMRI:* - Seed-based connectivity: Thalamus → cortex, DMN nodes - Graph theory metrics: Global efficiency, modularity, participation coefficient - Dynamic connectivity: Time-varying connectivity analysis. fMRI data will be preprocessed and analyzed following established best practices for functional connectivity analysis (Poldrack et al., 2011), including motion correction, spatial normalization, temporal filtering, and appropriate statistical thresholding procedures.

*DTI:* - Tract-based spatial statistics (TBSS) - Probabilistic tractography: Thalamocortical radiations - Microstructural metrics: FA, MD, RD, AD.

*EEG:* - Power spectral density (alpha, theta, beta bands) - Coherence analysis (thalamocortical, interhemispheric) - Source localization (sLORETA).

Primary Outcome: Correlation between thalamocortical connectivity and symptom severity.

Statistical Analysis: - Correlation: Connectivity metrics ~ symptom severity + reflex dysfunction - Mediation: Test if connectivity mediates reflex-symptom relationship - Group comparison: mTBI vs. controls on connectivity metrics - Multiple comparisons correction: FDR q < 0.05.

Expected Results (if model is valid): - Reduced thalamocortical connectivity in mTBI patients (Cohen’s d > 0.8) - Connectivity correlates with symptom severity (*r* > 0.5) - Reflex dysfunction mediates connectivity-symptom relationship.

#### Protocol 3: reflex-targeted intervention RCT

Objective: Test whether reflex-targeted therapy improves outcomes compared to standard care.

Design: - Study Type: Randomized controlled trial, parallel-group, assessor-blinded - Sample Size: *n* = 80 (40 per group; power analysis: 80% power to detect d = 0.6, *α* = 0.05) - Duration: 12-week intervention + 6-month follow-up - Inclusion Criteria: Persistent post-concussion symptoms (>3 months), documented reflex dysfunction - Exclusion Criteria: Severe TBI, psychiatric disorders, ongoing litigation. Study design follows established clinical trial methodology principles including randomization, blinding, and intention-to-treat analysis (Friedman et al., 2015).

##### Intervention groups

*Group 1: Reflex-Targeted Therapy (n = 40)* - Vestibular rehabilitation (2x/week, 45 min) - Gaze stabilization exercises - Balance training (dynamic, sensory integration) - Habituation protocols - Oculomotor training (2x/week, 30 min) - Smooth pursuit, saccades, vergence - VOR cancellation exercises - Cerebellar stimulation (optional: tDCS or rTMS).

*Group 2: Standard Care (n = 40)* - Symptom-triggered aerobic exercise (Buffalo Concussion Treadmill Test protocol) - Cognitive behavioral therapy for symptom management - Education on pacing and activity modification.

##### Assessments

*Baseline, 6 weeks, 12 weeks, 6 months:* - Primary: PCSS total score - Secondary: - VOR gain (vHIT) - Postural stability (SOT) - Cognitive function (ImPACT) - Quality of life (SF-36) - Return-to-activity status - Neuroimaging (subset, *n* = 20 per group).

Primary Outcome: Change in PCSS score from baseline to 12 weeks.

Statistical Analysis: - Mixed-effects ANOVA: Group × Time interaction - Effect size calculation (Cohen’s d) - Responder analysis: Proportion achieving ≥50% symptom reduction - Mediation: Test if reflex improvement mediates treatment effect.

Expected Results (if model is valid): - Reflex-targeted group: 40% greater symptom reduction vs. standard care - Treatment effect mediated by VOR gain improvement (>30% mediation) - Benefits persist at 6-month follow-up.

#### Protocol 4: parameter estimation and model validation

Objective: Estimate patient-specific model parameters and validate predictive accuracy.

Design: - Study Type: Model-based analysis of longitudinal clinical data - Sample Size: *n* = 100 patients with weekly assessments for 12 weeks - Data Requirements: Time-series data on symptoms, reflex metrics, activity levels.

##### Modeling approach

*Step 1: Parameter Estimation* - Use Bayesian inference to estimate patient-specific parameters - Prior distributions based on literature ranges (Table 1) - Likelihood function based on observed symptom trajectories - MCMC sampling (Stan or PyMC3).

*Step 2: Model Validation* - Split data: Training set (70%) and test set (30%) - Fit model to training data - Predict test set trajectories - Compare predicted vs. observed (R^2^, RMSE).

*Step 3: Sensitivity Analysis* - Identify which parameters most influence model behavior - Test robustness to parameter uncertainty - Determine which measurements are most informative.

Primary Outcome: Predictive accuracy (R^2^ > 0.6 on test set).

Statistical Analysis: - Bayesian model comparison (WAIC, LOO-CV) - Parameter identifiability analysis - Cross-validation (k-fold, leave-one-out).

Expected Results (if model is valid): - Model explains >60% of variance in symptom trajectories - Parameter estimates fall within physiologically plausible ranges - Individual differences in recovery explained by parameter variation.

##### Bayesian parameter estimation methodology

Parameter values will be estimated using Bayesian inference methods, treating the literature-derived ranges in Table 4 as informed priors (Gelman et al., 2013; Friston, 2010). The estimation procedure will follow these steps:

1. Prior Distributions: Beta distributions centered on Table 4 ranges with variance reflecting literature uncertainty. For example, α ~ Beta (mea*n* = 0.5, variance derived from reflex gain studies).
2. Likelihood Function: Constructed from empirical data including reflex assessments (VOR gain, OKN asymmetry, primitive reflex scores), symptom severity measures, and neuroimaging connectivity metrics. The likelihood quantifies how well specific parameter combinations predict observed data.
3. Posterior Estimation: Markov Chain Monte Carlo (MCMC) sampling using Stan or PyMC to obtain full posterior distributions for all 12 parameters simultaneously (Carpenter et al., 2017). Multiple chains with different initializations ensure convergence (Gelman-Rubin statistic < 1.1).
4. Uncertainty Quantification: 95% credible intervals for each parameter, capturing estimation uncertainty. Posterior predictive checks assess model fit by comparing simulated data from posterior draws to observed data.
5. Model Comparison: Bayes factors and leave-one-out cross-validation comparing NERD to alternative models (e.g., single-mechanism models, purely network-based models) to assess relative explanatory power (Vehtari et al., 2017). Information-theoretic approaches including AIC and BIC provide complementary model selection criteria that balance goodness-of-fit with model complexity (Burnham and Anderson, 2002).
6. Sensitivity Analysis: Examine how posterior distributions change with different prior specifications to ensure results are not overly dependent on initial assumptions.

This approach acknowledges that initial parameter ranges are theoretical starting points that will be refined as empirical data accumulates (Stephan et al., 2017). The Bayesian framework naturally handles uncertainty and provides a principled method for updating beliefs as new evidence emerges. Critically, this estimation procedure itself serves as a validation test: if the model cannot be fit to empirical data with reasonable parameter values, or if posterior distributions are uninformative, this would constitute evidence against the framework.

### Falsification criteria

The NERD model will be considered falsified if:

1. No correlation between reflex dysfunction and symptoms:
  - If VOR gain, SOT scores, and primitive reflexes do not correlate with symptom severity (*r* < 0.2, *p* > 0.05).
2. No connectivity-symptom relationship:
  - If thalamocortical connectivity shows no relationship with symptoms or reflex dysfunction.
3. No intervention benefit:
  - If reflex-targeted therapy shows no advantage over placebo or standard care (effect size *d* < 0.2).
4. Parameter estimates implausible:
  - If estimated parameter values fall far outside physiologically plausible ranges.
  - If parameters are not identifiable from clinical data.
5. Poor predictive accuracy:
  - If model predictions do not match observed trajectories (R^2^ < 0.3).
  - If model performs worse than simple baseline-symptom models.

### Validation milestones

We propose a phased validation approach:

Phase 1: Feasibility and Proof-of-Concept.

- Goal: Establish basic reflex-symptom correlations.
- Studies: Protocol 1 (longitudinal cohort).
- Success Criteria: Significant correlations (*r* > 0.3, *p* < 0.05).
  Phase 2: Mechanistic Validation.
- Goal: Confirm neuroimaging predictions.
- Studies: Protocol 2 (neuroimaging validation).
- Success Criteria: Connectivity patterns match predictions (effect size *d* > 0.6).
  Phase 3: Intervention Testing.
- Goal: Test therapeutic efficacy.
- Studies: Protocol 3 (RCT).
- Success Criteria: Significant treatment effect (*d* > 0.5, NNT < 5).
  Phase 4: Model Refinement and Integration (Years 5–7).
- Goal: Refine parameters, integrate with other models.
- Studies: Protocol 4 (parameter estimation), multi-site replication.
- Success Criteria: Predictive accuracy >60%, external validation successful.

### Model refinement strategy

Based on empirical findings, the model will be iteratively refined:

1. Parameter Adjustment:
  - Update parameter ranges based on estimated values.
  - Identify parameters that require re-conceptualization.
2. Structural Modifications:
  - Add missing nodes or connections revealed by data.
  - Simplify model by removing non-influential components.
3. Integration with Other Frameworks:
  - Incorporate metabolic dysfunction dynamics (Giza-Hovda model).
  - Integrate neuroinflammation cascades.
  - Link with neuropsychological models of symptom perception.
4. Computational Implementation:
  - Develop simulation tools for clinical use.
  - Create patient-specific prediction algorithms.
  - Build decision support systems for clinicians.

### Limitations

Current Limitations:

1. Theoretical Nature: The model is currently a theoretical framework without direct empirical validation, thus all predictions must be tested through rigorous controlled studies.
2. Simplified Dynamics: The model uses simplified differential equations that may not capture full complexity.
3. Parameter Uncertainty: Parameter ranges are estimates based on literature, not direct measurements.
4. Individual Variability: The model may not account for all sources of individual differences (genetics, premorbid factors).
5. Measurement Challenges: Some constructs (e.g., “network adaptability”) are difficult to measure directly.

## Clinical translation in context of treatment-resistant concussion

### Clinical context and unmet need

Persistent post-concussion symptoms (PPCS) represent a significant clinical challenge, with 10–30% of individuals experiencing symptoms lasting months to years after injury (Turner and LaBella, 2020; Mucha et al., 2022). Despite this substantial burden, there remains no consensus standard of care for chronic post-concussion rehabilitation (Hadanny and Efrati, 2016; Heslot et al., 2022). Systematic reviews consistently document heterogeneous, low-quality evidence for existing interventions, with many pharmacologic and non-pharmacologic treatments failing to demonstrate clear, reproducible efficacy (Moser et al., 2024; Ross et al., 2023). Randomized controlled trials comparing specialized interdisciplinary programs to usual care reveal that standard approaches often leave patients symptomatic (Rytter et al., 2019), and controlled studies of neuromodulation techniques report treatment resistance in chronic cases (Moussavi et al., 2019; Stilling et al., 2019). This evidence base underscores a critical gap: many patients with persistent symptoms exhaust conventional therapeutic options without achieving meaningful recovery.

### Development through clinical observation

The NERD framework presented here emerged from decades of clinical observation and iterative refinement in functional neurology practice. The foundational concepts trace to clinical work beginning in the late 1970s by Carrick and colleagues, who developed and refined approaches targeting hemispheric integration, vestibular-visual coordination, and sensorimotor rehabilitation in neurologically compromised populations (Carrick et al., 2015; Carrick et al., 2016; Carrick et al., 2017). These clinical observations - spanning over four decades of practice with diverse patient populations including traumatic brain injury, stroke, and persistent post-concussion syndrome - have informed the mechanistic principles and parameter ranges incorporated into the current model. Published case series and clinical trials emerging from this tradition have documented improvements in postconcussive symptoms, balance, and functional outcomes following targeted head-eye-vestibular-motion protocols and brain-based rehabilitation approaches (Carrick et al., 2015; Carrick et al., 2017). While such clinical observations are hypothesis-generating rather than definitive, they provide empirical grounding for the framework’s physiological assumptions and suggest clinical feasibility across multiple practitioners and settings.

### Preliminary empirical support

Preliminary empirical validation of interventions based on NERD principles is currently underway. A randomized controlled crossover trial testing multimodal rehabilitation targeting vestibular-visual integration and aerobic exercise in patients with persistent post-concussion symptoms has been completed and is currently under peer review (Jay et al., n.d.). This study, involving 25 patients with PPCS, compared a 4-week intervention combining visual and vestibular training with aerobic exercise against standard care, with subsequent crossover. Preliminary results suggest clinical feasibility and potential efficacy of approaches aligned with the mechanistic framework described here. However, these findings remain preliminary pending publication and independent replication.

### Balancing innovation and validation

The tension between clinical need and scientific rigor is particularly acute in PPCS, where patients often seek care after multiple failed interventions and where validated treatment protocols remain limited (Tapia and Eapen, 2017; Singh et al., 2018). The NERD framework offers a mechanistically-grounded, testable approach to understanding and addressing persistent symptoms. While comprehensive prospective validation remains essential - as outlined in the Validation and Testable Predictions section - the framework’s physiological basis, decades of clinical observation, and preliminary empirical support suggest it may inform individualized rehabilitation attempts in treatment-resistant cases. Such clinical application should occur within appropriate institutional oversight, with systematic monitoring of outcomes, and with clear communication to patients regarding the exploratory nature of the approach. The framework is best viewed as hypothesis-generating: it provides mechanistic rationale for rehabilitation strategies while acknowledging that definitive evidence of efficacy requires rigorous prospective trials. As validation evidence accumulates, the framework can be refined and clinical recommendations strengthened accordingly.

## Conclusion

The NERD model provides a mechanistically-grounded, testable framework for understanding persistent post-concussion symptoms. Emerging from decades of clinical observation in functional neurology practice and informed by established principles of systems neuroscience, the framework offers a structured approach to explaining the heterogeneity and persistence of symptoms that conventional models have struggled to address. We have outlined specific predictions, experimental protocols, and falsification criteria that will guide systematic empirical validation and enable rigorous testing of the model’s core hypotheses.

The framework’s value lies in its dual capacity: to structure future research through testable predictions and to inform clinical reasoning in cases where conventional approaches have proven insufficient. Preliminary empirical support from a randomized controlled trial testing interventions aligned with NERD principles (Jay et al., n.d.) suggests clinical feasibility, though these findings require independent replication and expansion. As validation evidence accumulates through the protocols outlined here, the framework can be iteratively refined, with clinical recommendations strengthened proportionally to the strength of empirical support.

For patients with treatment-resistant persistent post-concussion symptoms - a population for whom evidence-based options remain limited - the NERD framework may inform individualized rehabilitation attempts within appropriate institutional oversight and with systematic outcome monitoring. Such applications should proceed with clear communication regarding the exploratory nature of the approach and recognition that comprehensive validation is ongoing. The framework is best viewed as hypothesis-generating: it provides mechanistic rationale for rehabilitation strategies while acknowledging that definitive evidence of efficacy requires the rigorous prospective trials we have detailed. Ultimately, the NERD model aims to bridge the gap between clinical observation and mechanistic understanding, offering a path forward for both research and practice in a field where neither can afford to wait for the other.

## Data Availability

The original contributions presented in the study are included in the article/supplementary material, further inquiries can be directed to the corresponding author/s.
